# Method to Quickly Map Multifocal Pupillary Response Fields (mPRF) Using Frequency Tagging

**DOI:** 10.3390/vision8020017

**Published:** 2024-04-09

**Authors:** Jean Lorenceau, Suzon Ajasse, Raphael Barbet, Muriel Boucart, Frédéric Chavane, Cédric Lamirel, Richard Legras, Frédéric Matonti, Maxence Rateaux, Jean-François Rouland, José-Alain Sahel, Laure Trinquet, Mark Wexler, Catherine Vignal-Clermont

**Affiliations:** 1 Integrative Neuroscience and Cognition Center, UMR8002, Université Paris Cité, 75006 Paris, France; raphael.barbetv@gmail.com (R.B.); mark.wexler@gmail.com (M.W.); 2 Streetlab, 75012 Paris, France; suzon.ajasse@streetlab-vision.com; 3 CNRS, INSERM UMR-S 1172-Lille Neurosciences & Cognition, 59000 Lille, France; muriel.boucart@chru-lille.fr; 4 Institut des Neurosciences de la Timone-CNRS UMR 7289, 13005 Marseille, France; frederic.chavane@univ-amu.fr; 5 Hopital Fondation, Adolphe de Rothschild 29, rue Manin, 75019 Paris, France; clamirel@for.paris (C.L.); cvignal@for.paris (C.V.-C.); 6 LuMIn, CNRS, ENS Paris-Saclay, Centrale Supelec, Université Paris-Saclay, 91192 Orsay, France; richard.legras@ens-paris-saclay.fr; 7 Centre Monticelli Paradis d’Ophtalmologie, 13008 Marseille, France; frederic.matonti@free.fr; 8 Centre BORELLI, Université Paris Cité, ENS Paris-Saclay, CNRS, INSERM, SSA, 75006 Paris, France; maxence.rateaux@aphp.fr; 9 Department of Ophthalmology, Hôpital Claude Huriez, CHRU de Lille, 59037 Lille, France; jean-francois.rouland@chru-lille.fr; 10 Department of Ophthalmology, University of Pittsburgh, Pittsburgh, PA 15219, USA; j.sahel@gmail.com; 11 Faculté des Sciences Médicales et Paramédicales, Aix-Marseille Université, 13385 Marseille, France; laure.trinquet@univ-amu.fr

**Keywords:** pupillometry, frequency tagging, ophthalmology

## Abstract

We present a method for mapping multifocal Pupillary Response Fields in a short amount of time using a visual stimulus covering 40° of the visual angle divided into nine contiguous sectors simultaneously modulated in luminance at specific, incommensurate, temporal frequencies. We test this multifocal Pupillary Frequency Tagging (mPFT) approach with young healthy participants (N = 36) and show that the spectral power of the sustained pupillary response elicited by 45 s of fixation of this multipartite stimulus reflects the relative contribution of each sector/frequency to the overall pupillary response. We further analyze the phase lag for each temporal frequency as well as several global features related to pupil state. Test/retest performed on a subset of participants indicates good repeatability. We also investigate the existence of structural (RNFL)/functional (mPFT) relationships. We then summarize the results of clinical studies conducted with mPFT on patients with neuropathies and retinopathies and show that the features derived from pupillary signal analyses, the distribution of spectral power in particular, are homologous to disease characteristics and allow for sorting patients from healthy participants with excellent sensitivity and specificity. This method thus appears as a convenient, objective, and fast tool for assessing the integrity of retino-pupillary circuits as well as idiosyncrasies and permits to objectively assess and follow-up retinopathies or neuropathies in a short amount of time.

## 1. Introduction

Renewed interest in research on pupillary activity manifests in a tremendous increase in publications over the recent years. Owing to the particular circuits underlying pupillary dilation and constriction [1,2], two distinct fields developed, rooted in the seminal studies conducted in the 1960s (e.g., [3]). On the one hand, pupil constriction is controlled by a parasympathetic pathway originating from melanopsin-containing, intrinsically photosensitive, retinal ganglion cells (ipRGCs), a class of retinal ganglion cells (RGCs) that projects onto the pretectal olivary nucleus (PON). Neurons from the PON then project to the Edinger–Westphal nucleus (EW) that innervates the ciliary ganglions driving the sphincter muscle that constricts the iris. On the other hand, pupil dilation relies on a three-neuron sympathetic circuit originating in the hypothalamus, then projecting to the superior cervical ganglion, descending in the spinal cord and climbing along the internal carotid and the ophthalmic artery and ending in the pupillary dilator muscle.

As a consequence of these dichotomous circuits, publications fall into two categories: those concerned with pupillary dilation—mydriasis, mainly related to a sympathetic drive associated to cognitive functions—, and those focusing on pupillary constriction elicited by light—myosis, mainly involving the parasympathetic pathway—and mostly used in clinical studies to probe eye health. It is out of the scope of the present study to summarize the very numerous publications related to both fields, and several recent reviews are available to appreciate this renewed interest [4,5,6,7,8].

Table 1 presents the number of publications concerned with pupillary responses and diseases, illustrating the recent evolution of the field.

One reason for this increased interest relates to the discovery of intrinsically responsive retinal ganglion cells (ipRGCs containing melanopsin) [2,3] and to the identification of their circuits and functions [9,10,11]. ipRGCs represent 5% of ganglion cells whose main function is to drive pupillary responses to light through a dual activation: 1. by blue light (in the 480 nm range) through the action of melanopsin contained in ipRGCs’ bodies and dendrites, resulting in slow membrane potential depolarization that evokes slow modulations of pupil size (>1 s) adapting vision to ambient light; or 2. through the fast activation of photoreceptors, bipolar and amacrine cells [12,13,14] that project onto ipRGCs [10,15], resulting in fast (<1 s) modulations of pupil size (the pupil light reflex, PLR). 

Both the slow and fast circuits are impaired whenever ipRGCs are damaged, but defective photoreceptors also cause a dysfunction of the later fast circuit, which manifests by alterations of PLR characteristics [16,17]. Therefore, characterizing pupillary responses to light provide a simple, objective, and non-invasive way of assessing eye health, assuming that a defective pupillary response to focal light stimulation onto a particular retinal location is caused by a defect of either photoreceptors or ipRGCs in the corresponding retinal region. Importantly, ipRGCs share many structural and anatomical features with RGCs, including their sensitivity to similar harms [16]. Finally, damage to the optic nerve may also perturb pupillary dynamics [18,19].

In both the “Clinical” and “Cognitive” fields, with some exceptions [4,20,21], the focus is on transient pupillary responses, elicited either by flashes of light (PLR) or by timely controlled, cognitive tasks (involving attention, memory, decision, confidence, etc.) [6,22]. Recent studies tested different methods: continuous stimulation, with either full-field stimuli recruiting the whole retina [20], discrete focal light flashes [23,24,25], or sustained flickering luminance oscillations (Frequency Tagging). A recent study compared different pupillary tests stressing their advantages over Standard automated Perimetry (SAP) relying on subjective evaluations of the visibility of visual targets spread in the visual field [26,27].

### Sustained Multifocal Pupillary Frequency Tagging (mPFT)

In this dynamic context, we searched for a novel, convenient, and fast way of simultaneously assessing pupillary reactivity to light in different regions of the visual field so as to characterize a Pupillary Response Field, PRF, reflecting the *relative* contribution of multiple retinal locations to pupillary dynamics. Such PRF should permit to probe the integrity of the retino-pupillary circuits, and possibly to characterize the regional origins of visuo-pupillary defects.

We explain below the principle of a novel method relying on “Frequency Tagging”.

The reasoning underlying Frequency Tagging is that a periodic (sinusoidal) luminance modulation at a particular temporal frequency in a particular region of the visual field must entrain a sustained oscillatory pupillary activity at the same stimulation frequency if the modulation frequency is low (<5 Hz) and if the retino-pupillary circuits recruited by the visual stimulus are functionally healthy. A pupillary signal continuously recorded over time should display a possibly delayed oscillatory behavior at the visual stimulation frequency, which should correspond to a peak at the stimulation frequency in the spectral power of the recorded pupillary signal, associated to a phase lag (the delay between stimulus oscillations and pupillary oscillations). If several incommensurate, temporal frequencies, each coupled to a luminance modulation of a different region, are mixed, a multiplexed oscillatory pupillary activity corresponding to the combination of all the different temporal modulation frequencies composing the visual stimulus should again correspond to distinct power peaks at the stimulation frequencies.

We found that up to nine temporal frequencies associated to nine homogeneous non-overlapping regions could be mixed while still eliciting pupillary responses whose spectral power reflects the contribution of each frequency/region. With this approach, we could map “Pupillary Response Field” at once in less than a minute. Assessing a “Pupillary Response Field” simultaneously in different regions of the visual field presents several advantages,: it permits to compare the *relative* contribution of each of the retinal regions stimulated at once. It should thus be immune to sequential effects that could bias pupillary responses obtained at different times, as may be the case with sequentially presented light flashes. The *relative* power distribution is expected to be little altered by comorbidities, medications, or drugs that may nevertheless modulate the global pupillary responses, possibly reducing the absolute amplitude of pupillary oscillations. The *relative* spectral power may also reflect compensatory mechanisms if a frequency/region failed to trigger pupillary activity. In addition, using a continuous stimulation avoids the need to return to pupil baseline, as it is the case when measuring the PLR, such that all recorded data are relevant for analyses. Finally, a continuous stimulation permits to record and analyze eye movements and blinks made during sustained fixation, known to be altered in some ophthalmic and neurologic pathologies (e.g., [28,29,30]).

Below, we detail the methodology that was tested with 36 young healthy adults and present the data analysis workflow and the results. We then summarize additional experiments evaluating its robustness: test/retest, effects of light adaptation or of attention. We further analyze the correlation of pupillary spectral power with structural data (Retinal Nerve Fiber Layer, RNFL). We end with a summary of clinical studies [31,32] performed with mPFT on several neuropathies and retinopathies that demonstrate that mPFT reflects the location of retinal damages specific to each pathology and provides excellent classification results, opening the way to routine use in clinical settings. We then discuss this method with regard to current functional evaluations, as well as some limitations and advantages of mPFT.

## 2. Method and Stimuli

### 2.1. Stimulus

**Figure 1:**
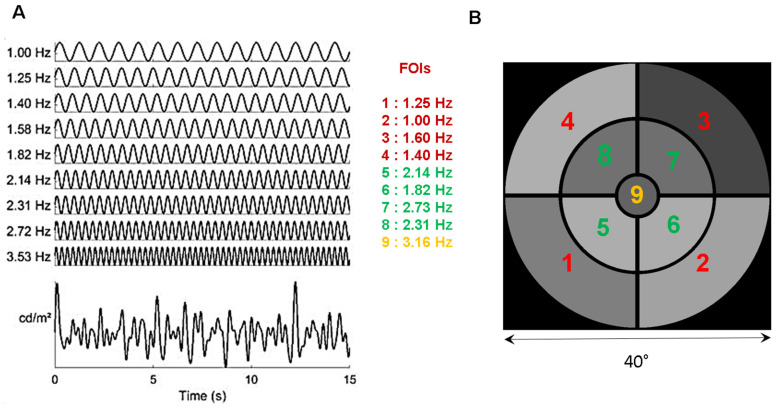
(**A**): Distribution of the temporal modulation frequencies (TMFs) and the resulting overall luminance modulation. (**B**). Stimulus configuration of the 9 sectors, each coupled with a TMF denoted by its index. The stimulus subtends about 40° of visual angle at 57 cm (central disk 4.6°; paracentral sectors, 5–19.6°; peripheral sectors 20–40°). See Video S1.

The stimulus consists of 9 sectors of different sizes, separated by black lines, overall covering about 40° of visual angle, arranged so as to stimulate central, paracentral, and peripheral regions (Figure 1). The sizes of the peripheral, paracentral, and central sectors are chosen on the basis of extensive preliminary experiments [33] so as to approximatively match the retinal magnification factor.

Each of the 9 sectors is periodically modulated in luminance at a specific temporal modulation frequency (TMF, Figure 1A), different for each sector and incommensurate with the other TMFs. Supplementary Video S1 shows an excerpt of the stimulus used in the study.

The temporal transfer function of the pupil, as assessed by Clarke et al. [34] in non-human primates, is low pass, with an upper limit of about 5 Hz, constraining the choice of the temporal frequencies that can be used to tag pupillary activity. The highest TMF in the stimulus must be lower than 4 Hz to ensure that reliable and sustained pupil responses are elicited during stimulation. The lowest TMF should be around 1 Hz to ensure that a too limited number of cycles during a run does not bias the spectral analyses. TMFs should be incommensurate so as to avoid the overlap of harmonics and fundamental frequencies as this could introduce artifacts and alter the analyses of the pupillary responses. In addition, care must be taken to avoid, as much as possible, TMFs corresponding to intermodulation products equal to the sum and difference between frequencies that could appear in the Fourier spectrum if non-linear interactions between frequencies were to occur. The 4 lowest TMFs are associated to the 4 eccentric annulus sectors; the 4 intermediate TMFs are associated to the 4 para-central annulus sectors. The highest TMF is presented in central vision to weight the otherwise dominant foveal contribution to pupillary activity [27]. As a result of these constraints, the chosen TMFs are 1.00, 1.25, 1.39, 1.58, 1.81, 2.14, 2.31, 2.73, and 3.16 Hz (Figure 1). The minimum amplitude of luminance oscillations and the minimum test duration that still evoke reliable pupillary responses at all Frequencies of Interest (FOIs) were assessed in preliminary experiments [34]. The shortest stimulation duration we found was about 45 s. The mean luminance of each sector, expressed in RGB units, is set to 127 (red, green and blue guns set to 127) and the modulation amplitude to 60 (in RGB units). With the screen settings used in the present study, this corresponds to a mean luminance of 51 cd/m^2^, a maximum luminance of 100 cd/m^2^, and a minimum luminance of 20 cd/m^2^. Note that the same stimulus is employed for the left and the right eye, with no attempt to balance the distribution of TMFs relative to the vertical meridian.

### 2.2. Apparatus

The stimuli displayed on a conventional monitor (Dell 2407WFPHC, 1024 × 768 × 8 bits refreshed at 60 Hz) placed at 57 cm from the eyes were generated by custom software (Jeda, Version 1.0) [35], running under Windows 10 (Microsoft Ltd., Redmond, WA, USA). Monocular eye movements and pupil size were recorded at 500 Hz with a Live Track Lightning remote eye tracker (Cambridge Research System Ltd., Rochester, UK, https://www.crsltd.com/tools-for-vision-science/eye-tracking/livetrack-lightning/, accessed on 15 January 2024) placed at 30 cm from the eyes. Recordings were down-sampled to 60 Hz for further analyses. A chinrest was used to stabilize the participants’ eye positions relative to the eye tracker.

### 2.3. Procedure

The session started with positioning the participants and checking the quality of the eye-tracker signals. A short 5-point calibration was performed once for the whole session (note that precise calibration is not required here, as the measure of pupil size is independent of calibration quality, and that relative eye movements are sufficient to analyze fixation (in)stability). A short questionnaire was administered to the participants to assess their general state (fatigue, existence of treatments, etc.) during which they adapted to the dim ambient light of the testing room. A session comprised 3 tests of 1 min each, presented to each eye, resulting in 6 min of recordings. One test was the mPFT grey test described herein. A second test used chromatic mPTF. The last test aimed to measure the Pupil Cycle Time (PCT) using a computerized setting [36]. We here report the results for the grey mPFT test only. For all these tests, participants were simply asked to maintain fixation at the center of the screen, marked by a small colored circular fixation disk (0.2°), and to limit, as much as possible, the number of blinks, with no other concurrent task (the fixation disk randomly changed color—red, green, blue, yellow—every 2 s, allowing for the addition of a task, if needed, such as counting the number of occurrence of a specific color). A brief rest between the different runs was used to change the stimulated eye (right or left in alternation). We evaluated test/retest variability in some participants (N = 8) by repeating the mPFT test 2 or 3 times on different days. Overall, a session lasted less than 15 min (note that running the grey mPFT stimulus for both eyes takes only 2–3 min).

A mPFT run started with the onset of a white centered fixation target, followed after 1 s by a brief full-screen flash (166 ms) at the maximum screen luminance (165 cd/m^2^) to elicit pupil light reflex (PLR). A dark screen was then displayed for 3 s. Afterward, the static mPFT homogeneous grey stimulus (51 cd/m^2^) was presented for 3 s to let the pupil adapt to the mean luminance, followed by 45 s of fixation of the temporal luminance modulations of the 9 sectors.

### 2.4. Participants

A total of 36 students (age range 20–29, 18 females) in the school of optometry of Université Paris-Saclay with normal or corrected to normal vision were enrolled in this study. All underwent the pupillary tests described above as well as Optical Coherence Tomography (OCT) and an ophthalmic examination (visual acuity) as part of their university course. All participants provided informed written consent in accordance with the Declaration of Helsinki. The study was approved by the local ethics committee “Comité de Protection des Personnes OUEST IV”, IRB #2020-A00859-30057.

### 2.5. Data Analyses

One data set from one participant was corrupted because of a technical issue and removed from further analyses. All remaining data were included in analyses performed with Matlab R2018b (The MathWorks, Natick, MA, USA). The raw pupil data, down-sampled at 60 Hz, were first corrected for blinks and recording artifacts. Blinks were detected as zeros in the pupillary traces. The corresponding blink data were replaced by a smoothed linear interpolation using a pre- and post-blink offset of 4 samples (66.6 ms). As the pupil is unlikely to change rapidly, we detected “artefactual transients” for which the pupillary signal was varying faster than a threshold set once for the whole data set, calculated using an ad hoc formula combining velocity and acceleration values of the pupil area. These “transient” data were replaced by a smoothed interpolation to limit their impact on the power spectrum computed afterward using the *fft* Matlab function.

As the initial and final pupillary recordings were sometimes noisy, and to allow for pupil entrainment at the beginning of a run, the beginning and end of the pupillary signal were trimmed by 60 samples (1 s), so that 43 s of pupillary responses were used in the analyses (2580 samples). The corrected pupillary signals were then z-scored; a ramping window of 100 samples, ranging from 0 to 1, was convolved with the beginning of the trace, and a window ranging from 1 to 0 was convolved with the end of the trace to prevent the abrupt onset and offset from introducing spurious power in Fast Fourier transform (FFT). Fourier analyses of the so-corrected pupillary signals were computed separately for each FOI. As a matter of fact, obtaining reliable amplitude and phase estimation for each FOI requires that the FOI to be analyzed is an integral multiple of the frequency resolution, FR (equal to the sampling frequency, SF, divided by the number of samples, N). When this condition is met, the FOI power does not spread across different bins of the spectrum, while it does spread across frequencies otherwise, biasing the estimates of power and phase. To meet this constraint for all FOIs, we adjusted the frequency resolution, FR, by decreasing the number of samples, N, FR = SF/(N − x), with x adjusted separately for each FOI so that FOI/FR was an integral value (or very close to an integral value, using a tolerance threshold equal to 10^−6^). Tests performed on the stimulus signal proved that this method was efficient and reliable to precisely recover both the signal powers and phases for each frequency. The amplitudes and phases of the pupillary signal were thus obtained after adjusting the FR for each FOI in this way. To estimate the phase lag between the stimulus and the pupillary signal, we first computed the phases of the luminance modulations of the stimulus itself for each FOI so as to take the temporal offset (60 samples) into consideration. We then computed the phases of the pupillary signals for each FOI. The differences between the pupillary phases and the stimulus phases, phase shift at each FOI were computed with the CircStat toolbox [37]. In addition, the cross-correlation between the average time-varying luminance modulation of the stimulus and the pupillary oscillations (after detrending the pupillary signal to suppress low frequency and linear drift components) was computed to derive the lag between both signals using the *xcor* Matlab function, thus providing an overall estimate of the time needed for retinal processing, propagation time through the optic nerve and relay nuclei, as well as the mechanical constriction and dilation time constants of the iris sphincter and dilator muscles.

We observed that the spectrum of the pupillary signal tends to follow a 1/f distribution (Supplementary Figure S1A) and further contains “noisy” components, possibly related to imperfect signal correction. We therefore decided to normalize the raw spectral powers computed for each FOI. We achieved this by dividing the mean of the spectral power at bins surrounding the FOI (SP(foi − 1)+ SP(foi) + SP(foi + 1))/3 by the mean of the spectral power in remote surrounding bins (SP(foi − 3) + SP(foi − 2) + SP(foi + 2) + SP(foi + 3))/4. Normalizing the power spectra in this way takes into account the spurious noisy power that can exist for each frequency bin while homogenizing the FOI power distribution. Several other normalization procedures (by frequency or by regional bins of the spectrum) were tested but appeared to distort or skew the FOI power distribution for some participants (Supplementary Figure S1B–D).

We ascertained that peaks in individual spectral power were triggered by and phase locked to the stimulus’ FOIs by comparing the spectral power of the pupillary responses averaged across all participants with the average of all individual power spectra. If individual pupillary responses were not phase-locked to FOIs, random phase shifts would flatten the mean pupillary response, whose power spectrum would lack peaks at FOIs, or at least would decrease their respective power. This was not the case (Supplementary Figure S2), demonstrating that luminance modulations did entrain the pupillary response for each FOI, although with varying power depending on the region at stake (see Results). Time–frequency maps were also computed (with m = 96) to verify that the power spectrum at FOIs was sustained during a run and did not result from short successive episodes of oscillatory activity at different times for different FOIs (Supplementary Figure S3).

### 2.6. Statistical Analyses

Test/retest variability was assessed in two ways: i. Pearson’s correlation coefficient and Bland–Altman plots [38] were computed with all FOIs; ii. Depending on the normality of the power distribution for each FOI, assessed with a one-sample Kolmogorov–Smirnov test, Student’s t tests or Mann–Whitney U tests were performed for each pair of data (Run1 and Run2 for each FOI).

Similar statistics were used to compare the effect of dark- versus light-adapted conditions.

We also computed Pearson’s correlation coefficients between the averaged retinal nerve fiber layer (RNFL) measured with Optical Coherence Tomography (OCT) and the averaged spectral power to evaluate whether the functional pupillary responses depend upon the structural characteristics of the participants’ retinae.

## 3. Results

All but one recordings of the 36 participants were included in analyses (70 eyes), despite some recordings having a large number of blinks or transient corrected data whose distributions are shown in Supplementary Figure S4 for all participants.

For each run, we computed 55 variables characterizing pupil and eye movement activity over time (Table 2).

**Figure 2:**
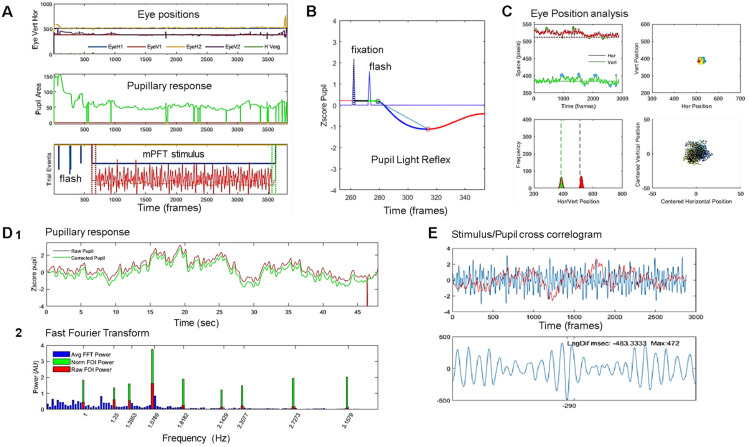
Steps of analyses: (**A**). Visual inspection of raw eye movements, pupillary activity, and technical event tracks. (**B**). Analysis of the PLR after blink detection and correction from which 5 descriptive variables are derived (see Table 1 for the list of all features derived from analyses). (**C**). Analysis of eye movements—fixation (in)stability—during the stimulation, from which 6 variables are computed. Top left: position over time; Top right: position over space, whole screen; Bottom right: zoom on centered spatial eye positions. Bottom left: histogram of vertical and horizontal eye positions. (**D**). (**1**) Raw (red line) and pupillary signal corrected for blinks and transient data (green line) during mPFT stimulation, with computation of 7 descriptive variables and characterization of 5 global pupillary variables, including stimulus/signal cross-correlation lag. (**2**) FFT of the corrected signal, estimating the amplitude spectrum: full spectrum (blue lines); Raw power at FOIs (red bars); Normalized FOI power (Green bars). (**E**). Cross-correlation between the stimulus luminance oscillations and the pupillary response. Top stimulus oscillation (blue line) and pupillary response (red line). Bottom, cross-correlogram results indicating the lag and correlation distribution between the stimulus and the pupil response.

Figure 2 presents an example of the different steps of our pipeline analyses that includes A. Visual inspection of raw eye movements, pupillary activity, and technical event tracks; B. Analysis of the PLR after blink detection and correction of transients from which 5 descriptive variables are derived (see Table 1 for the list of all features derived from analyses); C. Analysis of eye movements—fixation (in)stability—during the stimulation from which six variables are computed; D. 1. Delineation of the relevant pupillary signal recorded during the mPFT stimulation, correction of blinks and transient data, computation of seven descriptive variables, and five global pupillary variables extracted from the corrected pupillary signal; 2. FFT analyses estimating the amplitude and phase for each FOI (not shown); and E. Computation of stimulus/signal cross-correlation lag.

Figure 2D shows the results of the FFT transform of the pupillary signal. The whole FFT spectrum is shown in blue, and the raw (red bars) and normalized (green bars) pupillary spectral power (PSP) of the sustained pupillary response shows peaks at FOIs, indicating that the pupillary response was modulated by each of the nine stimulus sectors.

**Figure 3:**
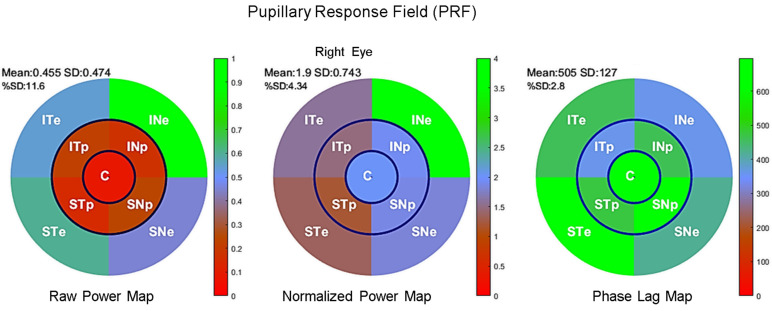
Maps for the right eye of an individual Pupillary Response Field for raw power (**left**), normalized power (**middle**), and phase lag (**right**) according to the sectors of mPFT stimulationlabelled according to its projection onto the retina (ST: supero-temporal; SN: supero-nasal; IN: infero-nasal; IT: infero-temporal. C: central; e: eccentric p: paracentral) and has a color reflecting its value relative to the color scale (right of each figure).

Pupillary Response Field maps of each participant’s eyes were derived from Fourier spectra and plotted for each sector with a color code indicating the raw, normalized power, and phases for each of the nine FOIs. Figure 3 shows an example of the PRF of the left eye of a single participant, indicating the mean and standard deviation of the power distribution (top left numbers). Numbers displayed on each sector indicate the TMF, FOI power, and the percentage of pupillary activity relative to the sum of all FOI power of each sector.

### 3.1. Distribution of Pupillary Spectral Power

**Figure 4:**
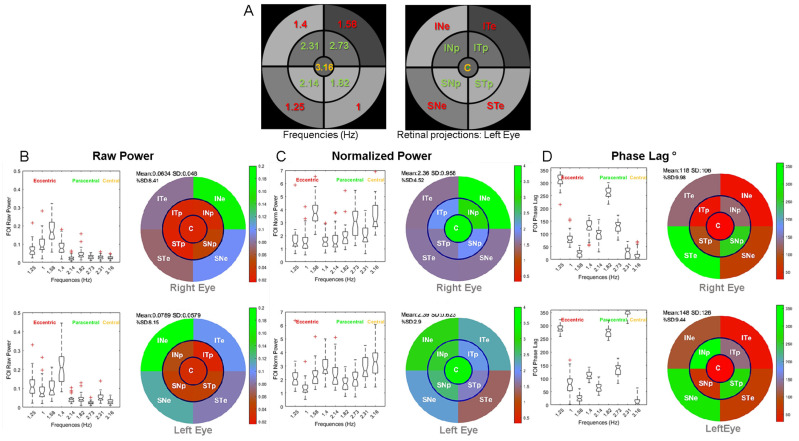
Group results showing the power distribution (boxplots) and Pupillary Response Fields (PRFs) of the right and left eyes: (**A**): FOI distribution (Hz) and retinal projections of sectors for the left eye. Labels for each sector are as in Figure 2. (**B**): Distribution of raw power for the 9 FOIs and associated PRFs for the right (upper panel) and left (bottom panel) eyes. (**C**): Distribution of normalized power for the 9 FOIs and associated PRFs. (**D**): Distribution of phase lags for the 9 FOIs and associated PRFs.

Figure 4 shows the group results for the right and left eye. Figure 4A shows the correspondence between the stimulus sectors and the associated FOIs (Hz). Figure 4B shows the distribution of raw power for the nine FOIs and associated PRFs. Figure 4C shows the distribution of normalized power for the nine FOIs and associated PRFs. Figure 4D shows the distribution of phase lags for the nine FOIs and associated PRFs after correction for outliers.

The power distributions and multifocal Pupillary Response Fields (mPRF) in Figure 4 show that the pupillary spectral power (PSP) is heterogeneous across sectors, and hence across the visual field, with a larger raw power for peripheral sectors than for paracentral sectors for the right and left eyes (*p* < 0.00001 for all eight Student *t* tests). Similarly, the spectral power of the central sector was significantly lesser than that of each of the eccentric sectors (*p* < 0.0001 for all eight Student *t* tests). Finally, the spectral power for the sector projecting onto the infero-nasal retinae (INe) was significantly larger than that of each of the other eccentric sectors (*p* < 0.001 for all *t* tests). The distribution of PSPs and phase lags was, however, similar across individuals, indicating that Pupillary Response Fields computed with our method reflect a common spatio-temporal organization of retino-pupillary dynamics, despite idiosyncrasies. This homogeneity at the group level further suggests that short episodes of fixational instability or blinks that differ amongst individuals did not significantly perturb the sustained pupillary response during a run (see Figure 10 and Discussion below).

One possible account of the PSP differences across sectors relates to the different TMFs used for each sector, rather than to the sectors’ locations in the visual field. To address this question, we performed additional analyses of the present data, as well as a control experiment in which two very different TMF distributions were used (see Supplementary Figure S5). The fact that the power distribution differs in the right and left eyes despite the TMFs being similarly distributed (the same stimulus was used for the right and left eyes) argues against a pure contribution of TMFs. To test this statement, we took advantage of this TMF distribution to compare the raw power for each sector of both eyes, either using identical TMFs corresponding to different retinal sectors’ projections or using identical sectors’ retinal projections that are coupled to different TMFs. In the latter case, the distribution of power of the left eye was flipped around the vertical meridian so that the sector’s retinal projections in both eyes coincided. Eight pairwise Student *t* tests were computed to compare the z-scored PSP of eccentric and paracentral sectors of each eye. We found that eight out of eight *t* tests were significant (*p* < 0.01) when comparing the power for each TMFs, but that only 4 of the 8 *t* tests were significant when comparing the power of each sectors’ retinal projections (*p* < 0.05). The later significant *t* tests corresponded to the paracentral sectors (Supplementary Figure S5A). The fact that the PSP differences were significant in both analyses for the TMFs coupled with the paracentral sectors suggests that PSP differed in the left and right eyes. In the control experiment (with different participants and slightly different settings), we used two different TMF distributions: i. the one used here, with “low” TMFs coupled to eccentric sectors and the “high” TMFs coupled with the paracentral sectors, and ii. an inverted distribution where the “low” TMFs are coupled with the paracentral sectors and the “high” TMFs are coupled with the eccentric sectors. Our analyses of both TMF configurations again indicate that the distribution of PSP is mainly related to the sectors’ retinal projection (Supplementary Figure S5B,C). Altogether, these results suggest that, with the range of TMFs used here for the eccentric and paracentral sectors, from 1 to 2.72 Hz, temporal frequency *per se* does not fully account for the power distribution across sectors, although TMF may contribute to some extent to PSP distribution.

One exception is the raw power observed for the central disk sector. It is known that light stimulation within the macula elicits large pupillary responses compared to paracentral and peripheral stimulations [27]. This large pupillary response to central stimulation is further corroborated by a dense distribution of ipRGCs around the macula [39]. Here, the central region is coupled with the highest TMF, 3.17 Hz, for which the temporal transfer function of the pupil starts to decline [34]. As a matter of fact, our choice of coupling the highest TMF to the central region was dictated by this decreased response to “high” temporal frequency so as to limit the contribution of macular stimulation to the overall pupillary activity, which could otherwise limit or dominate the contribution of other regions [27].

Overall, the spatial distribution of PSP appears to reflect the intrinsic pupillary sensitivity of different retinal regions. In line with studies showing that the infero-nasal retina, on which the upper temporal sector projects, we found larger pupillary responses for infero-nasal stimulation as compared to other, non-foveal regions [23,24,27]. This finding corroborates the non-uniform distribution of human ipRGCs characterized by their greater density in the infero-nasal retina [39].

A complementary point to note is that mPFT is mostly designed to characterize retino-pupillary defects in individuals with ophthalmic diseases. So, all things being equal, the comparisons between the distributions of PSP in healthy subjects with those of patients for a given spatio-temporal distribution of TMF and sector sizes and eccentricity is what may inform us on the existence of an ophthalmic condition.

### 3.2. Timing of Pupillary Oscillations

Of interest is the distribution of phase lags presented in Figure 4D, as it could reveal retino-pupillary processing delays, reflecting retinal temporal integration, propagation times through the optic nerve, and nuclei lying between the retina and the iris muscles (see [1]), as well as the mechanistic time constant of iris motility. Although the phase lags shown here are very different across sectors, they are very similar across participants after correcting for outlier phase values. The lag distributions for the right and left eye are similar, indicating that phase lags are related to TMFs, independently of the sector locations. These distributions are irregular (in terms of phase and delay), which likely relates to the circular nature of phase processing with FFT (evaluated modulus 2kπ). Correcting these values to match the hypothesis that excessively short or excessively long phase delays are unlikely requires several assumptions regarding the expected timing of pupillary responses. We preferred not to consider that the computed phase lags reflect veridical physiological lags, as some values are at odds with reports of PLR latencies but may still be relevant for assessing differences between healthy individuals and patients. In our view, the phase lags computed herein indicate that the mPFT stimulus did entrain TMF-dependent pupillary oscillations in a similar way for all participants.

**Figure 5:**
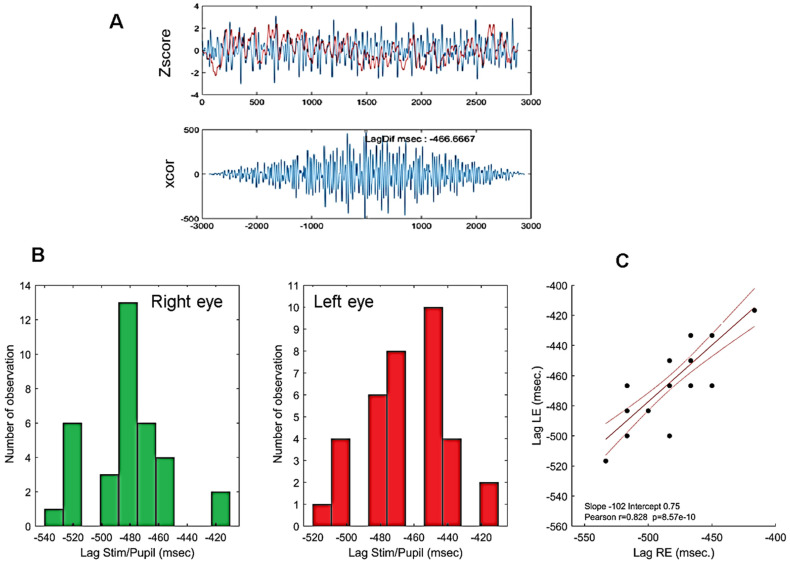
(**A**): Example of an individual stimulus/pupillary signal cross-correlation using the Matlab *xcor* function. (**B**): Histogram of phase lags for all participants. Bottom left: right eye; bottom middle: left eye. (**C**): Correlation between cross-correlation lags of the right and left eyes of all participants. Red lines show the linear regression (r = 083, *p* < 0.0001) together with 95% confidence intervals. Note that because pupillary signals are down-sampled to 60 Hz, the time resolution of lags is only 16.666 ms such that phase lags from different participants overlap. The high correlation shown here indicate that similar lags are observed for the right and left eyes of each participant.

Computing stimulus/signal cross-correlation lags seems to be a better way of assessing the overall latency of the pupillary responses relative to the mPFT stimulus. The temporal lags (computed with the *xcor* function of Matlab for each run) are around 500 ms (range 420–540 ms, Figure 5) and are comparable to the maximum constriction latencies reported for the PLR, ranging between 500 and 600 ms [23,40,41].

**Figure 6:**
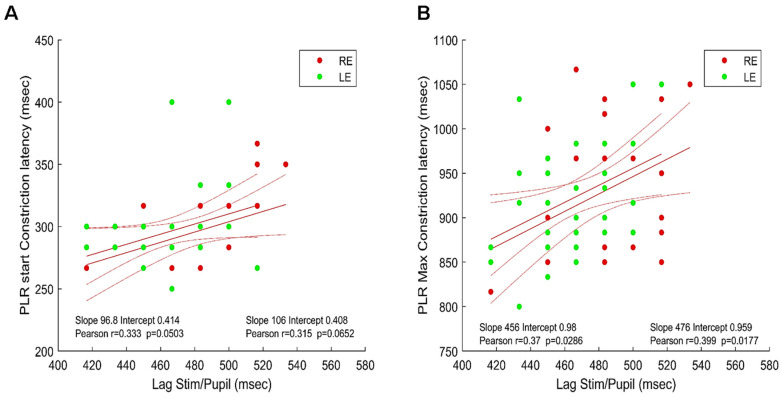
Maxima of the cross-correlations between the stimulus/signal (phase lags) and PLR latencies. (**A**). Lag vs. PLR start constriction latency: right (red disks) and left (green disks) eyes. (**B**). Lag vs. PLR maximum constriction latency: right (red disks) and left (green disks) eyes. Red lines show the linear regressions for the two eyes together with 95% confidence intervals Inserts indicate the values of Pearson’s coefficient correlation for each eye.

The extent to which the latencies of the PLR recorded at the beginning of a run (see Method and Table 1) and the stimulus/signal cross-correlation lags are correlated is shown in Figure 6.

Figure 6A shows the latency of the beginning of pupil constriction elicited by a full-field flash (see Method) as a function of the stimulus/pupil cross-correlation lag during sustained stimulation; Figure 6B shows the latency of the constriction peak as a function of the cross-correlation lag during sustained stimulation.

As it can be seen in Figure 6, the better and significant correlations are obtained between the stimulus/pupil lag and the latency of the maximum PLR constriction for both eyes. This suggests that the measure of the stimulus/pupil lag does reflect a relevant aspect of the timing of pupillary responses during sustained stimulation, capturing some of the inter-individual differences found with PLR. Note that the average stimulus/signal lags (482.4 and 463.3 ms for the right and left eyes, respectively) are shorter, but still in the range of the PLR latencies reported in the literature (about 550 ms in [23] or [40]) despite very different stimulation conditions.

Also, note that the latency of maximum constriction of the PLR is computed after dark adaptation such that pupil baseline size is at its maximum. In contrast, the lag computed with a sustained mPFT stimulus reflects the continuous adaptation of pupil size to varying luminance intensities, mixing dilation and constriction, which could account for the differences reported here.

### 3.3. Test/Retest with mPFT

Determining whether mPFT is reliable and stable is important if it were to be routinely used for clinical assessments. To evaluate the repeatability of mPFT, several participants (N = 8) repeated the protocol two times on different days and at different times.

We first evaluated repeatability by comparing the pupillary features extracted during data analyses for each participant and at the group level. Pearson’s correlation coefficients and Bland–Altman plots between Run1 and Run2 were computed for raw power, normalized power, PLR and global pupillary variables (see Table 1 for a detailed list).

**Figure 7:**
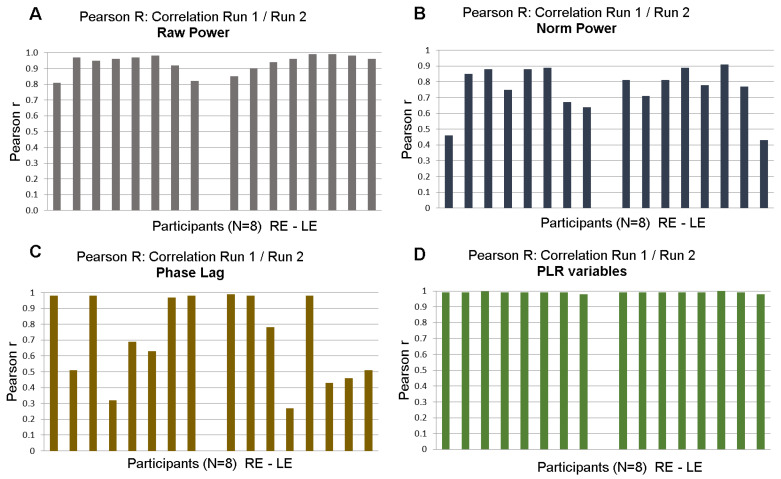
Test/retest: distribution of Pearson’s r coefficient between Run 1 and Run 2 of 8 participants for right and left eyes. (**A**). Correlations for raw power; (**B**). Correlations for normalized power. (**C**). Correlations for phase lags. (**D**). Correlations for PLR variables.

Figure 7 shows the distribution of Pearson’s correlation coefficients for each of the eight participants for different variables (raw and normalized power, phase lags and PLR). With few exceptions for raw and normalized power, test/retest correlations were high for all participants. Correlations for the phase lags were not as large, presumably because phase lags were computed modulo 2kπ.

**Figure 8:**
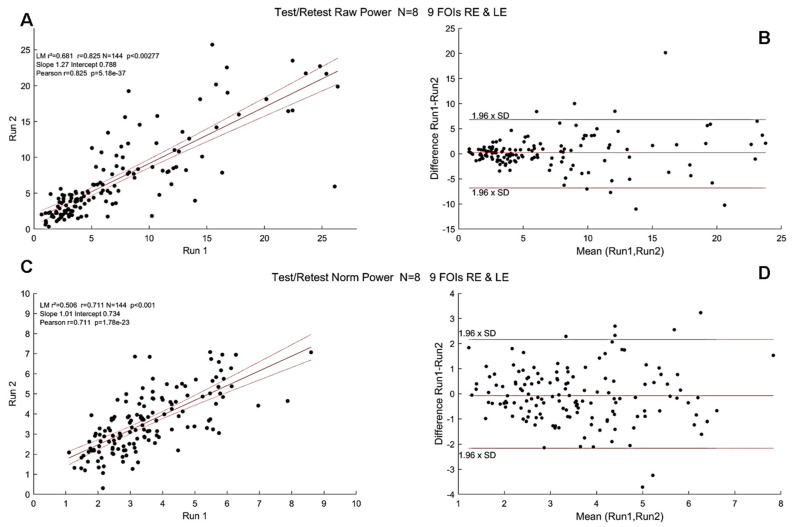
Test/Retest Pearson’s r coefficient at the group level (N = 8, pooled right and left eyes). Black dots show the PSP for all FOIs. (**A**). Correlations for raw power; (**B**). Bland–Altman plot for raw power. (**C**). Correlations for normalized power. Red lines show the linear regression together with 95% confidence intervals. (**D**). Bland–Altman plot of normalized power. Horizontal lines show the 95% confidence intervals.

Finally, Figure 8 shows correlations of raw (r = 0.82) and normalized powers (r = 0.71) at FOIs and Bland–Altman plots at the group level for raw and normalized powers.

These test/retest analyses indicated that the variables derived from mPFT are stable over time. It can be noted, however, that correlations are better for raw compared to normalized FOI power, presumably because the power normalization performed here takes into account the residual power of the FFTs (see Data Analyses) that may vary depending on the corrections of blinks and transients that could introduce small artifacts resulting in power spreading over all frequency bins. Correlation values are smaller for the phase lags, presumably because the estimates of some phase lags are biased by the circular nature of this variable (see above the section “Timing of pupillary oscillations”).

In addition to these analyses, we compared the power for each FOI in Run1 and Run2. After determining that the power distributions were not normally distributed (One-sample Kolmogorov–Smirnov test), we computed Mann–Whitney U tests. All tests—one per FOI in Run 1 and 2—were not significant (all *p* > 0.05), indicating that the power distribution of each FOI did not significantly differ during the two runs.

### 3.4. Correlations between Spectral Power and RNFL Thickness in Young Healthy Participants

**Figure 9:**
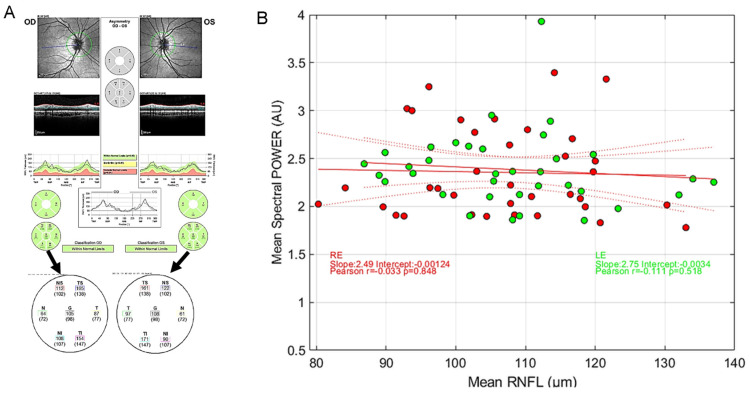
(**A**) Example of RNFL data and extraction of relevant values from a PDF file. (**B**) Distribution of the average mPFT power as a function of the average RNFL values for the right eye (red symbols) and the left eye (green symbols). Red lines show the linear regression together with 95% confidence intervals. No correlation is found between the two variables. (inserts show Pearson’s correlation coefficients, with different colors for the 2 eyes).

Several clinical studies report correlations between pupillary features of the PLR and Retinal Nerve Fiber Layer (RNFL) estimated with Optical Coherence Tomography [20,31,32,42,43,44]. In these studies, depending on the protocol and stimuli at stake, different features of pupillary signals from populations of patients with ophthalmic pathologies are measured and compared to structural characteristics of the retina. To our knowledge, no such correlation has been looked for in a cohort of young healthy participants to evaluate the existence of functional/structural correlations. In order to perform such comparison, we first decoded the RNFL values from the PDF files that summarize the OCT results (Spectralis, Heidelberg Engineering, Heidelberg, Germany) of each participant and plotted the averaged RNFL values as a function of the averaged spectral power (Figure 9).

As it can be seen in Figure 9, the averaged RNFL varies from about 80 µm to more than 130 µm in this population of young healthy participants. Similarly, the averaged mPFT powers differ across individuals. However, we found no significant correlation between the averaged RNFL and averaged mPFT power. This suggests either that the thickness of the RNFL is not determinant for the functioning of the pupillary circuits, at least in the observed range for this population, or the fact that comparing the average values of RNFL and mPFT power is irrelevant because the means do not capture the intra-individual variability of each distribution across the retina. We attempted to refine this analysis by comparing the mean RNFL values of the upper retina (TS, NS, see Figure 9A) with the mPFT power of the lower sectors, and the lower RNFL retinae values (TI, NI) with the mPFT power of the upper sectors. These comparisons did not reveal significant correlations between the two variables, possibly because the retinal location of these measures (RNFL and spectral power) does not closely coincide.

So, contrary to clinical studies conducted on some pathologies (glaucoma in particular) showing correlations between RNFL and pupillary responses [20,32,42,43], the same does not hold for young healthy subjects. We hypothesize that RNFL/spectral power correlations can be found only for extreme values (very thin RNFL for advanced pathologies), but not necessarily in young healthy subjects.

### 3.5. Effects of Blinks and Eye Movements and of Recording Quality

**Figure 10:**
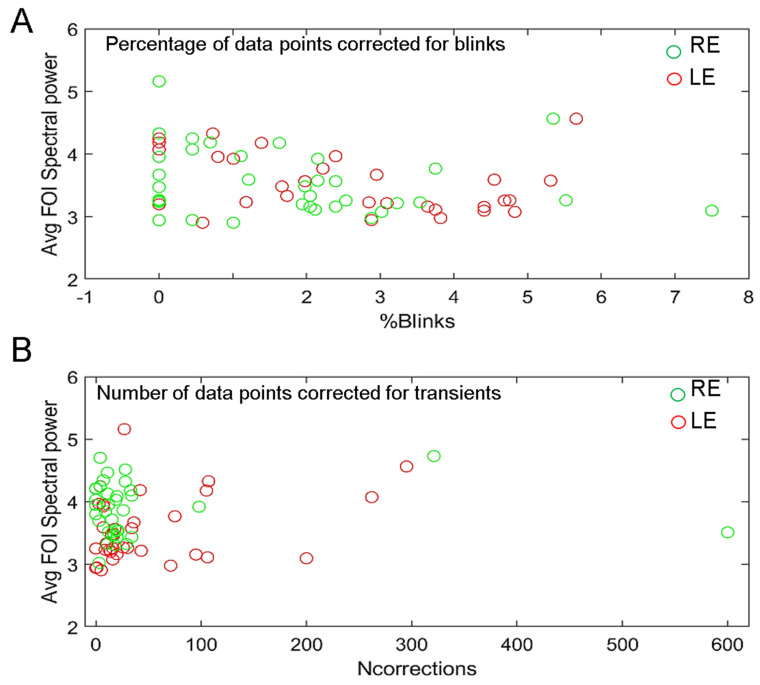
(**A**). Averaged spectral power of each participant as a function of the percentage of blink-corrected data for the right (green symbols) and left (red symbols) eyes. (**B**). Averaged spectral power as a function of the number of data corrected for transients for the right (green symbols) or left (red symbols) eyes.

One may wonder whether the correction of blinks and transient data that are replaced by smoothed linear interpolations before the FFT computation (see Method) influences the overall power at FOIs. To verify this possibility, we plotted the percentage of blink-corrected data and the number of transient corrected data as a function of the average spectral power of each participant (Figure 10). This comparison did not reveal any strong correlation between the two variables, as the averaged power of some participants with little or no corrected data is sometimes less than that of participants who make many blinks.

### 3.6. Effects of Attention on mPFT Spectral Power

As mentioned in the introduction, focused attention and other cognitive factors induce a pupillary dilation, although of modest amplitude [6,45,46] through activation of the sympathetic pathway which in turn sends inhibitory projections onto the Edinger–Westphal nucleus driving the pupil [1]. Could attention modulate and bias the spectral power of mPFT stimulation? Although attentional lapses or focused attention onto a sector could modulate pupillary activity, these pupillary modulations are unlikely to counterbalance the continuous strong and sustained visual drive of mPFT. As a matter of fact, attentional modulations of pupil size are mostly found when luminance variations are small (e.g., [45]). For attention to significantly modulate pupillary activity during mPFT, sustained covert attention to one or several sectors would be needed during the whole test, which appears difficult to perform, and therefore unlikely. To nevertheless test for this possibility, we conducted experiments with young healthy participants (N = 16, different from those who participated in the present study) whose task was to count the number of times two colored disks displayed side by side in one sector (the same sector during a run) had the same hue. The colors of the disks changed every second (with 25% of similar hue for both disks). Each of the 45 s trials (one for each sector, except the central disk, resulting in eight trials) thus required covertly attending to a single location during the whole duration of the test, with a task involving counting and memory, both being cognitive tasks known to entrain a pupil dilation (see, e.g., [6]). A control condition used the same stimuli, but with no associated task (passive fixation). The experiment was performed with two luminance modulation amplitudes of the nine sectors (large, as used herein, vs. half the amplitude modulation used here) to determine whether covert attention is more likely to influence the spectral power when the visual drive is reduced. We did find a significant increase in the mean spectral power in the attention condition relative to the no task one (Student *T* test, *p* < 0.02) that was larger for a smaller visual drive (Cohen’s d test: 0.44 for large luminance modulation vs. 0.75 for the lower luminance modulation). However, we did not find significant selective increases of spectral power associated to the sector where the colored disk stimuli were displayed. As we used a visual stimulus—colored disks superimposed to the sectors—that could itself modulate pupil size, we performed an additional experiment using auditory signals in which participants had to count the number of sounds with a higher pitch (460 Hz) than a reference (440 Hz, with 25% of sounds with a higher pitch). This experiment did not reveal any significant effect of the auditory task on the averaged mPFT power (Student *T* test, *p* > 0.05).

Thus, sustained attention does modulate the average mPFT spectral power, but this effect appears global and not spatially selective, and it is larger with a smaller visual drive such that attention does not appear to be able to significantly modulate the distribution of *relative* power. Note that the two tasks were demanding and difficult to perform. It is unlikely that similar attentional effects would affect the spectral power when the task is simply to look ahead at the center of the mPFT stimulus and when the amplitude of the luminance modulations is large, as used herein.

### 3.7. mPFT and Dark Adaptation

In most studies using the PLR to probe a clinical condition, one difficulty is to evaluate pupillary responses relative to a well-defined baseline. This necessitates defining a period during which no event that could alter pupil size occurs. In research, each study can use an arbitrarily defined baseline, provided it is well characterized in terms of luminance and duration, to allow for reproducibility. When considering clinical pupillometry, the conditions defining the baseline must be universal to allow for comparisons between healthy and unhealthy conditions worldwide. The current consensus is to measure pupil size after dark adaptation, for duration lasting from 5 to 20 min [8]. This is very constraining if the goal is to devise tools to be routinely used to probe a clinical condition, as it requires a significant amount of time. In addition, each flash of light inducing a pupillary response must be followed by a period allowing pupil size to return to baseline, in the order of 3 to 10 s, although alternative approaches have been proposed (e.g., [24,44]).

Unfortunately, dark-adapting patients before testing is constraining and long, at odds with the needs in overbooked clinical services. Moreover, obtaining reliable PLR data may require repeating the test several times, resulting in long-lasting sessions.

Dark adaptation may not be necessary with mPFT, because the sustained multipartite stimulation may still reveal the *relative* imbalance of reactivity to light across the visual field, whatever the adapting level. In addition, mPFT stimulation may be more similar to the ecological situations encountered by individuals in everyday life, where continuous modulation of light intensities is common and thus more likely to reveal functional defects of retino-pupillary dynamics.

To test whether the adapting level deeply changes the absolute and relative mPFT spectral power, we performed an experiment to compare mPFT power distribution after dark adaptation (10 min, 0–6 lux) or after adapting to day light (4000 to 7000 lux for 30 to 60 s before each run). This experiment was performed with a single subject repeating the test many times (N = 42 trials, 21 runs per condition). In the light-adapted condition, the mPFT test was ready to launch with a simple mouse click so that the time elapsed between exposure to day light and the beginning of the test, conducted in a dark room, was less than 30 s. We used Student *t* tests to compare the FOI power distributions in both conditions. We found that the averaged raw spectral power was different in the two conditions (t = −5.32, *p* < 0.001), but not the averaged normalized power (t = −0.117, *p* = 0.9). The normalized spectral power values of each FOI were not significantly different (all t < 2, all *p* > 0.07, Supplementary Figure S7). Some other significant differences were observed: the lag between the pupillary signal and the stimulus was longer in the light-adapted condition (−568 vs. −543 ms, t = −11.05, *p* < 0.01), and the slope of the linear fit computed on pupillary signals was significantly steeper in the light-adapted condition (t = 2.28, *p* = 0.02), suggesting that adaptation to the stimulus mean luminance developed during the test after day light exposure. Although more data should be collected on more observers to confirm these preliminary observations with very different day light and dark adaptation, these data suggest that long-lasting dark adaption prior to running the mPFT test may not be mandatory to identify visual defects in patients.

To verify whether our statement that the *relative* spectral power is more stable and reliable across conditions than the *absolute* spectral power (see above and Supplementary Figure S6), we compared the *absolute* and *relative* spectral power distributions in the dark-adapted and light-adapted conditions by computing the Pearson’s coefficient correlations between the two conditions (dark-adapted vs. light-adapted) for *absolute* power and *relative* power distributions. We found a correlation of r = 0.863 (*p* < 0.0001) between the *absolute* spectral power distributions and a correlation r = 0.928 (*p* < 0.0001) between the *relative* power distributions, suggesting that, indeed, the *relative* power distribution captures between-sector regularities not related to the differences of *absolute* power between the two conditions.

### 3.8. Summary of Clinical Studies Performed with mPFT

We used mPFT in several clinical studies to evaluate the extent to which it allows classifying healthy participants from individuals with ophthalmic diseases [31,32]. In addition, questionnaires evaluated the acceptability, comfort, duration, glare, fixating difficulty, and fatigue induced by mPFT.

In the first study [31], three rare pathologies were tested at the Paris Vision Institute: Retinitis Pigmentosa (N = 14), Stargardt disease (N = 14), and Leber hereditary optic neuropathy (N = 9). Healthy participants (N = 14) were also included. For this study, we used an EyeLink II eye tracker (SR Research, Ltd., Ottawa, ON, Canada) and the stimuli were back-projected on a large translucent screen (55.5 × 41.63 degrees of visual angle) while participants sat 128 cm away from the screen. The mPFT stimulus subtended an angle of about 40°.

The second study performed in the Ophthalmology Department of Lille [32], involved glaucoma patients (N = 28) at different stages of the disease as well as healthy participants (N = 17). A LiveTrack Lightning eye tracker (Cambridge Research Systems, Ltd., Rochester, UK) was used; the stimuli were presented on a computer screen (Dell 210-AXKQ) while participants sat 80 cm away from the screen.

Finally, we used mPFT to test patients with Age-related Macular Degeneration (AMD, N = 43), Diabetic Retinopathy (DR, N = 40) and Age-related Maculopathy (ARM, N = 13) together with healthy controls (N = 50) in a study conducted at the Monticelli Clinic in Marseille. In this study, a LiveTrack Lightning eye tracker (Cambridge Research Systems, Ltd., Rochester, UK) was used; the stimuli were projected on a computer screen (SyncMaster 2443, Samsung, Gyeonggi-do, Republic of Korea) placed in a cabin to control for ambient illumination, while participants sat 75 cm away from the stimulation screen.

For these different studies performed on different sites using slightly different setups and protocols (each including other pupillary tests), we used the same pipeline analysis as the one described herein.

**Figure 11:**
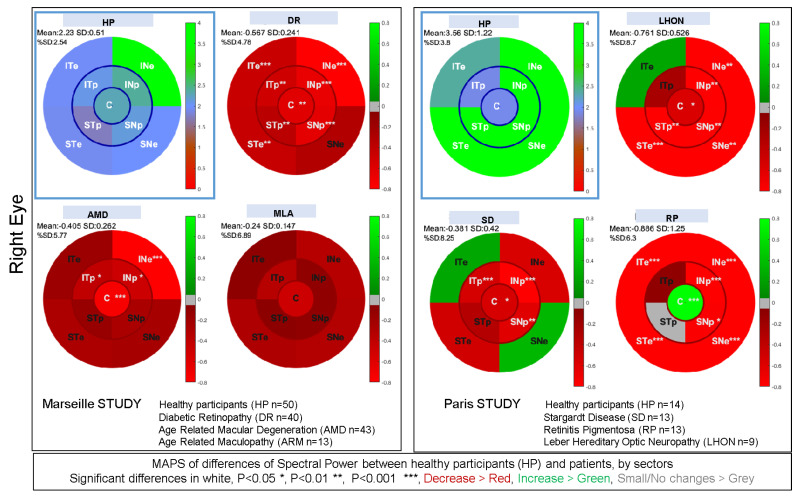
Maps of differences of spectral power between healthy participants and patients in the Marseille and Paris studies for each sector of the stimulation of the right eye (see Supplementary Figure S9 for the left eye). The reference maps of healthy subjects are framed with a blue square. The remaining maps present the differences of power relative to healthy participants for each of the studied pathologies: Age-Related Macular Degeneration (AMD), Diabetic Retinopathy (RD) and Age-Related Maculopathy (ARM) for the Marseille study; Retinitis Pigmentosa (RP), Stargardt disease (SD) and Leber Hereditary Optic Neuropathy (LHON) for the Paris study. Stars within each sector indicate the significance level (*p* < 0.05 = *; *p* < 0.01 = **; *p* < 0.001 = ***), written in white font. See text for details.

To estimate whether the pupillary power reflects regional defects, we computed the differences in power between healthy participants and patients for each sector. We further calculated the significance of these differences (Student *t* tests). We performed these calculations for the Marseille and Paris studies separately, as different settings were used in the two studies. Figure 11 shows the maps of regional differences for the right eye together with the *t*-test significance (see Supplementary Figure S9 for the left eye). As it can be seen, these maps reflect regional decreases in pupillary power characteristics of the pathologies at stake. In the Marseille study, we observed a general decrease in pupillary spectral power for Diabetic Retinopathy. For AMD, the central sector has a significantly decreased activity, in line with the damage of central vision found in these patients, although the PSP is also lesser for one eccentric sector (INe). For Age-Related Maculopathy (ARM), an early stage of AMD, the regional PSPs are not significantly different from those of healthy subjects for the right eye, but the PSP decreases significantly in the central sector in the left eye (see Supplementary Figure S9). Despite the small number of ARM patients included in this study, a general decrease in pupillary activity can nevertheless be observed. In the Paris study, we find large significant differences for peripheral sectors in Retinitis Pigmentosa, accompanied by spared responses for the central sector, and even a significantly increased response for that sector in the right eye. The distribution of these differences concords with the loss of peripheral vision characteristic of this disease. For Stargardt disease (SD), significant decreases in power are observed in the central and paracentral sectors, in line with the loss of central vision in these pathologies. In Leber hereditary optic neuropathy (LHON), sectors with significant PSP differences cover a large part of the visual field, including the central sector, in line with the characteristics of this disease.

In both studies, we sometimes found significant increase in power for some sectors (e.g., significant increase in PSP for the central sector of the right eye in Retinitis Pigmentosa). We speculate that these augmented responses reflect the fact that the damaged regions contribute less to the overall pupillary response, hence reducing the competition between sectors and allowing for a larger central contribution. Overall, these findings suggest that each pathology is characterized by a specific pattern of pupillary responses relative to that of healthy individuals, possibly providing individual bio-signatures of a disease.

To test this idea further, we computed the area under the curve of receiving operating characteristics (AUC of ROC) and the corresponding sensitivity and specificity using the *fitglm* and *perfcurve* functions available in Matlab (with a binomial distribution and logit link function in the linear regression model). AUCs of ROC were computed with the power and phase lags of the nine FOIs. We also used the *relative* power between sectors, computing the ratio of powers of up versus down and left versus right sectors, the ratios of peripheral and paracentral sectors, and the ratio of the central versus all other sectors (resulting in 12 values, see Supplementary Figure S6). We also included additional variables (e.g., global pupillary variables, see Table 1) derived from the pupillary traces to compute the AUC of ROC. Although these other variables do not provide information on the regions susceptible to malfunctioning, they may nevertheless characterize pathological conditions and improve the classification of patients and healthy observers.

When only the spatio-temporal information related to mPFT (FOI Power and Phase) are used for classification, all AUC of ROC computed for each disease of each study are above 0.8. All AUCs of ROC are above 0.95 when more variables are included. In these later cases, the gain in classification is mainly related to the global pupil state variables and to the number of corrections (% blink data and correction of transients, see Table 1). AUCs of one were obtained for both eyes and all diseases in Studies 1, 2 and 3, depending on the type and number of variables used for classification. In Study 3, an AUC of ROC~1 is still obtained when pooling the results from all the patients. Combining the data from the right and left eyes resulted in somewhat lower values (AUC > 0.8). In addition, distributions of mPFT power also allowed for classifying different diseases with AUC > 0.9.

Although these results indicate that pupillary responses elicited with mPFT permit to classify patients and healthy participants with excellent sensitivity and specificity, the number of participants included in each study remains relatively small, limiting the conclusions that can be drawn from these studies.

In addition, correlations between structural (RNFL obtained with OCT) and functional pupillary responses of mPFT were found in two studies [31,32].

Comparing the mPFT data of the present study with those of Study 3 performed on a different site (Marseille) with older healthy participants (mean 69 years, see above), different experimenters, and slightly different settings, we obtained somewhat comparable results, although the differences for the central and one paracentral sectors were significant (Supplementary Figure S8). These regional differences may relate to the age of the participants and reflect a genuine decrease in pupillary reactivity in the macula with aging, although differences in study settings may also play a role.

## 4. Discussion

The mPFT method presented here allows for objectively assessing multifocal Pupillary Response Fields reflecting the functional integrity of retino-pupillary circuits in a short amount of time (~1 min per eye). This method permits to analyze both the power and phase lag for each FOI and the overall latency of sustained pupillary responses to continuous luminance oscillations, together with several other variables, including global pupil size and reactivity, number of blinks or fixational eye movement stability. Test/retests performed on different days on a subset of participants and comparisons between studies conducted independently on different sites indicated that the regional distribution of mPFT power is stable over time, suggesting that mPFT is robust.

Importantly, the Pupillary Response Fields permit to analyze the *relative* contribution of each sector to the overall pupillary response recorded at once, and not only the *absolute* power for each sector, allowing to identify sectors with relatively less power than others, an important feature in the perspective of using mPFT with patients presenting localized visual defects (scotoma). Analyzing *relative* spectral power differences between sectors renders mPFT less prone to the influences of exogenous factors (e.g., ambient luminance, time of the day) and endogenous factors (medication, fatigue, or age, for instance) that may modulate the pupillary response. However, we did not test our subjects in these conditions, or after receiving mydriatic drugs currently used to examine the fundus of the eyes. We doubt that mPFT will still give reliable data with these drugs, which would impose that Pupillary Response Fields are recorded before or independently from an ophthalmologic examination requiring dilated pupils.

Although we did not extensively present and analyze the distribution of *relative* spectral power herein, we did compute 12 ratios between sectors or groups of sectors that can be used in clinical studies, as described in Supplementary Figure S6. As a matter of fact, using this distribution of spectral power ratios to sort patients with an ophthalmic disease from healthy participants did improve the classification results in our clinical studies [31].

The use of a continuous sustained stimulation further permits the evaluation of additional variables collected during a single run such as the number of blinks, the fixational instability, and “spurious” eye movements (saccades) known to vary with clinical conditions and thus relevant to assess the existence of ophthalmologic issues.

### 4.1. Visual Field Perimetry, Pupillary Response Fields, and Structural Retinal Imaging (OCT)

Do Pupillary Response Fields and Standard Automated Perimetry (SAP) probe similar, possibly defective, functional or structural features?

No clear answer to this question is yet available. SAP employs low-luminance targets of different sizes (<64 mm^2^) to estimate threshold values relying on subjective reports, while mPFT uses larger luminance modulations with extended sectors to elicit pupillary responses. mPFT relies on a dynamic stimulus changing luminance over time while SAP uses static targets presented in succession in different locations of the visual field. The visual field tested with SAP is larger (−30°: +30°) than that tested with mPFT (−20°: +20°). Furthermore, the circuits driving image-forming and non-image-forming visions (photo-entrainment and PLR) are different [47]. The outcomes of these different methods can therefore be very different. However, we found good agreements between the two methods when comparing Visual Field and Pupillary Power maps of glaucoma patients, althougyh we also observed discrepant results [32]. Interestingly, mPFT also allows for assessing processing times through the retino-pupillary circuits, a feature not available with SAP. The timing of pupillary response may bring additional information on the existence and effects of a clinical condition on vision, as was found in ROC analyses. For instance, damages to the optic nerve, such as the demyelination observed in optic neuritis, alter the temporal dynamics of pupillary responses [19].

As RGCs, ipRGCs driving pupillary responses are ganglion cells and may thus be sensitive to harms similar to those targeting RGCs, although their number, functioning, and distribution across the retina are different [7]. Evidence for a loss of ipRGCs in glaucoma is associated with pupillary changes proportional to disease severity [43], but better knowledge of the temporal and spatial development of visual, structural, and pupillary defects is needed.

The photoreceptors to ipRGC pathway drives pupillary responses faster than the intrinsic response of ipRGCs to blue light [11]. It is likely that this pathway is recruited by the mPFT stimulation and drives the oscillatory pupillary behavior. Diseases affecting cones or rods may thus alter the Visual Fields and Pupillary Response Fields in a similar way, although recent findings on knock-out mice indicate that image-forming and non-image-forming systems rely on ON and OFF circuits differently [47].

Several studies reported correlations between pupillary and structural markers in several pathologies [4,43,48,49]. We also found correlations between RNFL and pupillary mPFT power in retinopathies and neuropathies [31,32], but did not observe structural/functional correlation in the population of young healthy participants in the present study.

Alternately, even if Pupillary Response Fields would bear little resemblance with Visual Field Perimetry or with structural features seen with OCT, they may nevertheless characterize a functional defect, possibly signaling the existence of a clinical issue specific to the underlying pupillary circuits. Whether or not this malfunctioning entrains or correlates with a visual complaint (migraine, glare, over-blinking, blur) needs further investigation, but several studies found significant correlations between these symptoms and pupil reactivity to light [50,51]. Finally, we speculate that defects specific to the pupillary circuits (in the Edinger–Whestphal nucleus, for instance) may be identified with mPFT. For example, Scinto et al. [51] reported a selective cell loss in the Edinger–Whestphal nucleus in Alzheimer patients and proposed that pupillary hypersensitivity in Alzheimer disease may be caused by abnormalities in the Edinger–Whestphal nucleus (also, see [52,53]).

### 4.2. Eye Trackers and Use of mPFT

We tested mPFTs with a few eye trackers endowed with different characteristics: Eye-Link II (SR Research, Ottawa, ON, Canada), Livetrack Lightning (Cambridge Research Systems, Rochester, UK), EyeTribe and SMI Red250 (Sensory Motoric Instrument, Teltow, Germany). mPFT gave good results (clear distinct spectral power peaks at FOIs) with EyeLink II and LiveTrack Lightning, but not with EyeTribe or Red 250 from SMI. Independently, in the lab, Mark Wexler developed the mPFT stimulus and tested it with a PupilLabs eye-tracker (PupilLabs, https://pupil-labs.com/, accessed 15 January 2024, Berlin, Germany), successfully isolating peaks at FOIs in his pupillary response. Informal tests with Tobii Pro 500 (Tobbi, Ltd., Danderyd, Sweden) and the E(ye)BRAIN eye tracker developed by Suricog (https://www.suricog.fr/, accessed 15 January 2024, Paris, France) indicated that pupillary responses to mPFT also contained significant power at FOIs.

These tests were conducted with commercially available monitors (at 60 Hz) with different sizes and settings and in several lightning conditions or eye–screen distances (hence covering slightly different retinal regions) while still exhibiting well-behaved spectral power at FOIs in healthy individuals.

### 4.3. Limitations and Future Directions

Although the mPFT settings used here elicit reliable pupillary data that are sufficient to perform excellent classification of healthy participants and patients, improvements in the stimulation are possible. Factors that could be adapted concern size, luminance modulation amplitude, and coupling of TMFs with sectors. One limitation of the present settings is the spatial extent of the stimulus (about 40° of visual angle) that may be too small to detect visual defects in the far periphery that can signal the onset of a disease, as it is often the case for glaucoma. Simply reducing the eye–screen distance would permit to overcome this issue.

Another limitation is the spatial resolution of mPFT, as each sector used herein covers a rather large region of the visual field. We did test a version of mPFT with smaller sectors distributed in two opposite quadrants of the visual field, but, although similar classification results were found in Study #1 [31], two runs are needed to cover the whole visual field, thus lengthening the test duration. Because the nine sectors of the mPFT stimulus are large, mPFT may not be able to detect subtle and small focal visual defects which can be detected with other techniques such as multifocal ERG, for instance [54].

A third limitation is the use of an achromatic stimulation, as chromatic pupillometry develops and provides interesting results related to the retinal circuits involving ipRGCs, and is also relevant to characterize visual defects differently altering rods and cones [11,12,13,44].

A fourth limitation is related to eye movements. Indeed, if participants move their gaze and fixate different locations of the stimulus at different times, the retinal projections will vary accordingly, biasing the distribution of FOI power. Although similar issues arise with other functional tests, and despite the fact that we analyzed eye movements, we did not attempt to take these data into account to correct or adjust for this factor. Analyzing time–frequency maps in conjunction with fixational eye movements could permit identification and quantification of the effects of eye movements.

Finally, the clinical studies summarized in this article involve patients whose pathology is already well characterized, sometimes at an advanced stage. Using a pupillary test to follow-up these patients may not be that useful, thanks to the progress of retinal imaging, but may be relevant to evaluate the effects of treatments on pupillary responses in longitudinal studies. One interesting but challenging use of mPFT would be to screen for ophthalmic pathologies in populations at risk who could develop a yet unnoticed pathology, as it is the case in AMD or glaucoma. To that screening aim, a very large data set and the use of deep-learning appears necessary, and could help determining whether pupillary responses have the potential to early signal and identify retinal or neuronal issues, such as RNFL thinning.

## 5. Conclusions

We presented a novel method to map multifocal Pupillary Response Fields (PRFs) in a short amount of time with little burden for the participants that permits to distinguish healthy individuals from patients in a variety of neuropathies and retinopathies with excellent sensitivity and specificity.

This method is easy to use, not requiring a dedicated expertise to pass the test, relies on reflexive objective physiological signals, and could be used to complement the current functional examination performed in clinical services (Standard Automated Perimetry, SAP, in particular). Notably, this method may be used without the need for prior dark adaptation, an interesting feature if it were to be used in clinical settings. In addition, mPFT can be used with individuals unable to understand the instructions to perform a subjective evaluation (SAP), such as elderly with cognitive impairments, children, individuals not mastering the local spoken language, and possibly non-human primates.

Assessing malfunctioning retino-pupillary circuits is of interest in itself. It could reveal impairments not seen with other functional tests and bring additional information related to the physiopathology of the underlying retinal defects. Moreover, malfunctioning retino-pupillary circuits may entrain visual discomfort (e.g., for glaucomatous patients; see, for instance [55]). Indeed, the loss of ipRGCs that occurs in some diseases [20,55] degrades the pupillary reactivity to light, which could in turn cause glare in some lighting conditions.

Finally, the use of sustained stimulation permits recording and analyzing eye movement instability as well as blinks, known to also reflect the existence and severity of ophthalmic diseases [28,56,57].

To conclude, mPFT appears as a convenient and fast way of assessing defects in retino-pupillary circuits, although additional studies are needed to determine the extent to which its outcomes are altered by fatigue, medication, and underlying medical conditions, and to evaluate whether it can detect subtle and focal defects or relates to structural defects not significantly altering visual perception, as can be the case at an early stage in glaucoma patients.

## Figures and Tables

**Table 1:** Number of publications related to pupillary activity and diseases. Search with PubMed using different terms. This table lists all publications over time and publications released before and after 1999.

Search for Publications with PubMed Using the Words:					
Pupillary Disease		Disease PLR		Glaucoma Pupillary	
1869–2024	6611 results	1982–2024	2731 results	1876–2024	2580 results
1869–1999	2351 results	1982–1999	14 results	1876–1999	1014 results
2000–2024	4262 results	2000–2024	2717 results	2000–2024	1569 results
pupillary retinal disease		ophthalmic pupillary		retinopathy pupillary	
1947–2024	2129 results	1813–2024	20,971 results	1944–2024	2059 results
1947–1999	600 results	1813–1999	8272 results	1944–1999	593 results
2000–2024	1530 results	2000–2024	12,973 results	2000–2024	1467 results

**Table 2:** List of the variables derived from the analysis of each run.

Variables Derived from Each Pupillary Trace Recorded during a Run (for Each Eye)						
Pupil Light Reflex (PLR)	Global Pupillary Characteristics during mPFT Stimulation	Data Correction	Eyemovements	Raw and Normalized Spectral Power	Phase Lags	Relative Spectral Power
Pupil size during base line	Slope of mean pupil size over time (Pupillary Escape)	% of corrected blink data	Median vertical and horizontal positions	Raw Power values for the 9 FOIs	Phase lag values for the 9 FOIs	Left/Right Ratios by sector: 4 values
Start constriction latency	Mean pupil size	Number of corrected data	Number of outlier data (>2 SD from mean position Vertical & Horizontal)	Normalized Power values for the 9 FOIs		Up/Down Ratios by sector: 4 values
Peak constriction	Mean amplitude of pupillary oscillations		Correlation between pupil and eye-movements (Vertical & Horizontal)		Overall Lag (crosscorrelation of stimulus and pupillary signal)	Up/Down and Left/Right Ratios 2 values
Latency of peak constriction	Standard deviation of pupillary oscillations					Peripheral/Paracentral Ratio
Constriction speed						Central/Paracentral+Peripheral Ratio

## Data Availability

The data are available from the corresponding author, J.L., on reasonable request. The Matlab scripts can be shared with whoever is interested, and the Jeda software used for stimulation is available for download on request.

## Supplementary Materials

The following supporting information can be downloaded at: https://www.mdpi.com/article/10.3390/vision8020017/s1. Video S1: Excerpt of the mPFT stimulus; Figure S1: FFT and normalization; Figure S2: mPFT and phase locking; Figure S3: Time-frequency map; Figure S4: Distribution of corrected data; Figure S5: TMFs vs. sectors PSP distribution; Figure S6: Computation of relative PSP; Figure S7: PSP and dark/light adaptation; Figure S8: Comparison of PSP in young and old healthy adults; Difference between healthy participants and patients for the left eye; Figure S9: Maps of differences of spectral power between healthy subjects and patients for the left eye.
