# Supplementary figures and images for: Method to Quickly Map Multifocal Pupillary Response Fields (mPRF) Using Frequency Tagging
Source: Vision (Basel). 2024 Apr 9;8(2):17. doi: 10.3390/vision8020017 (PMC11036301; doi:10.3390/vision8020017)

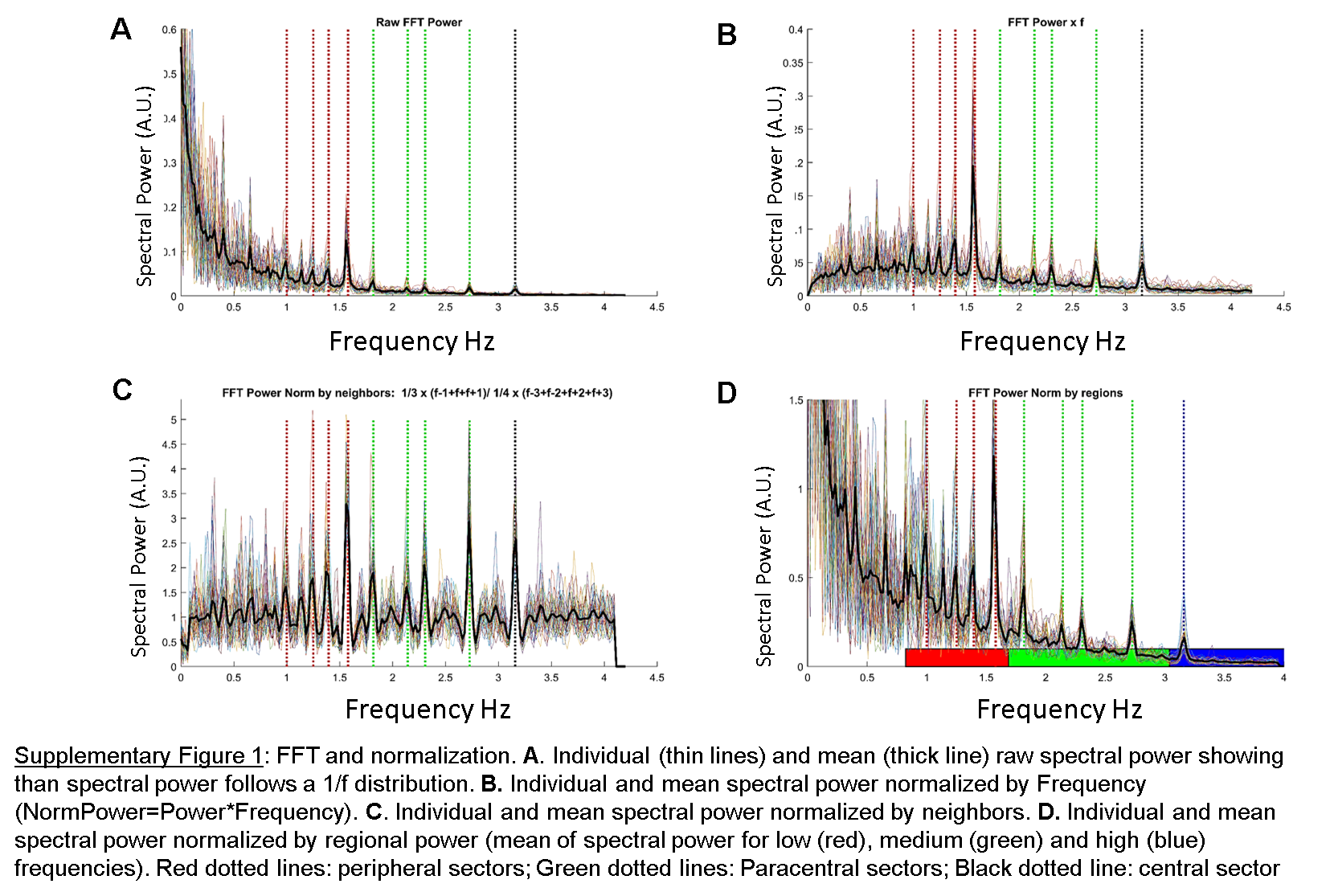

Supplement: Supplementary file 1 [file vision-08-00017-s001.zip › Supplementary Figure S1.png]

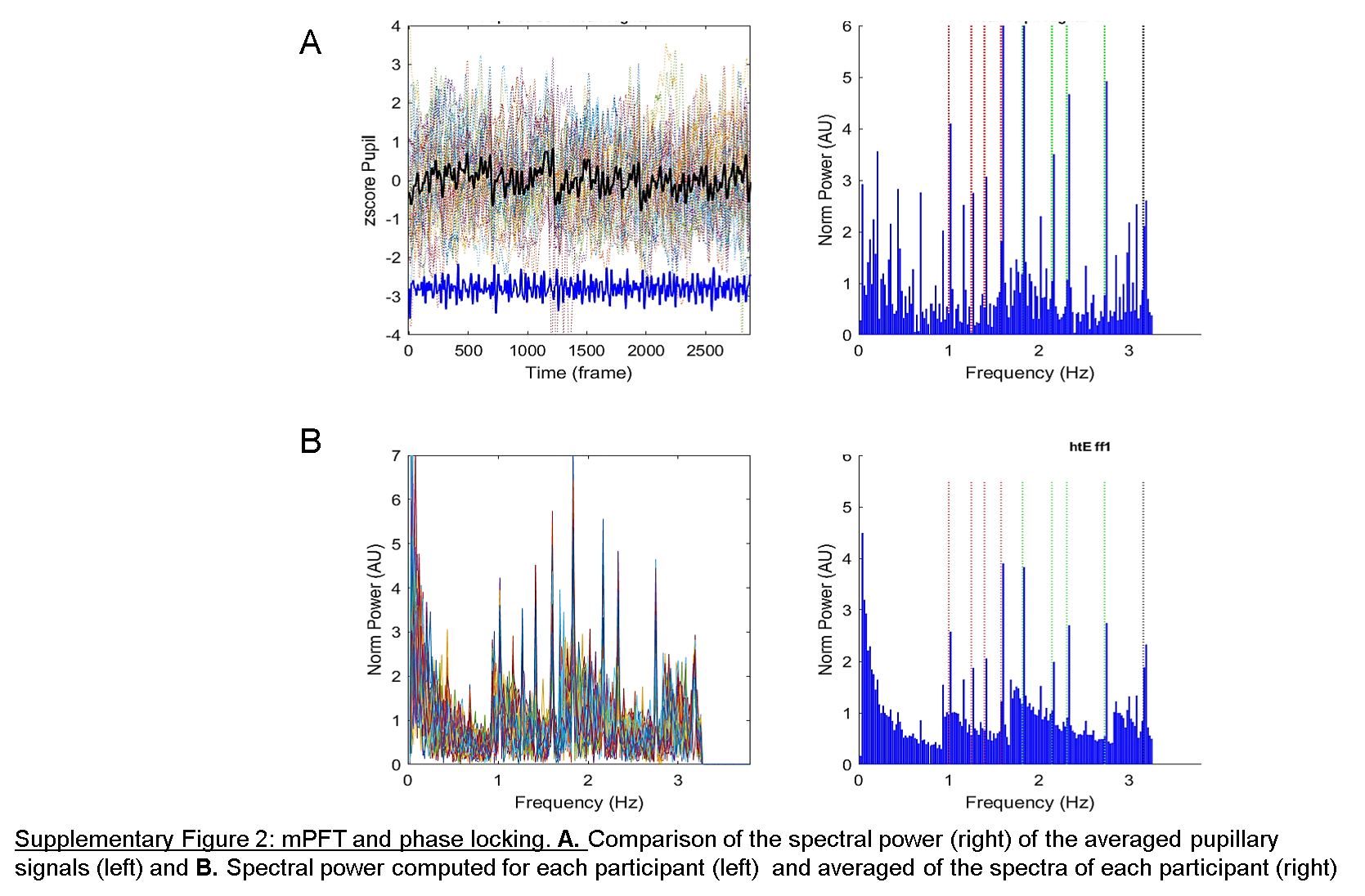

Supplement: Supplementary file 1 [file vision-08-00017-s001.zip › Supplementary Figure S2.png]

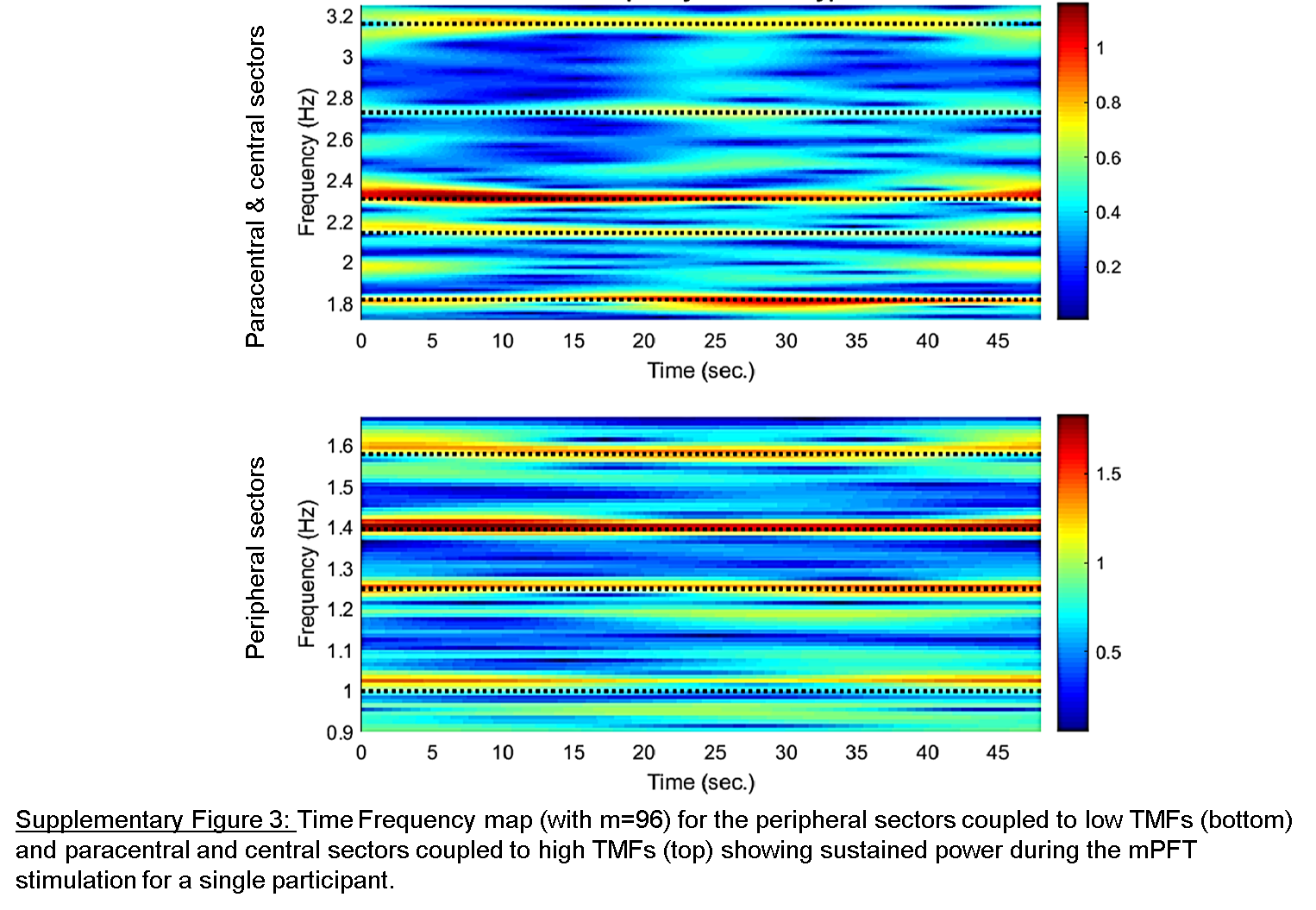

Supplement: Supplementary file 1 [file vision-08-00017-s001.zip › Supplementary Figure S3.png]

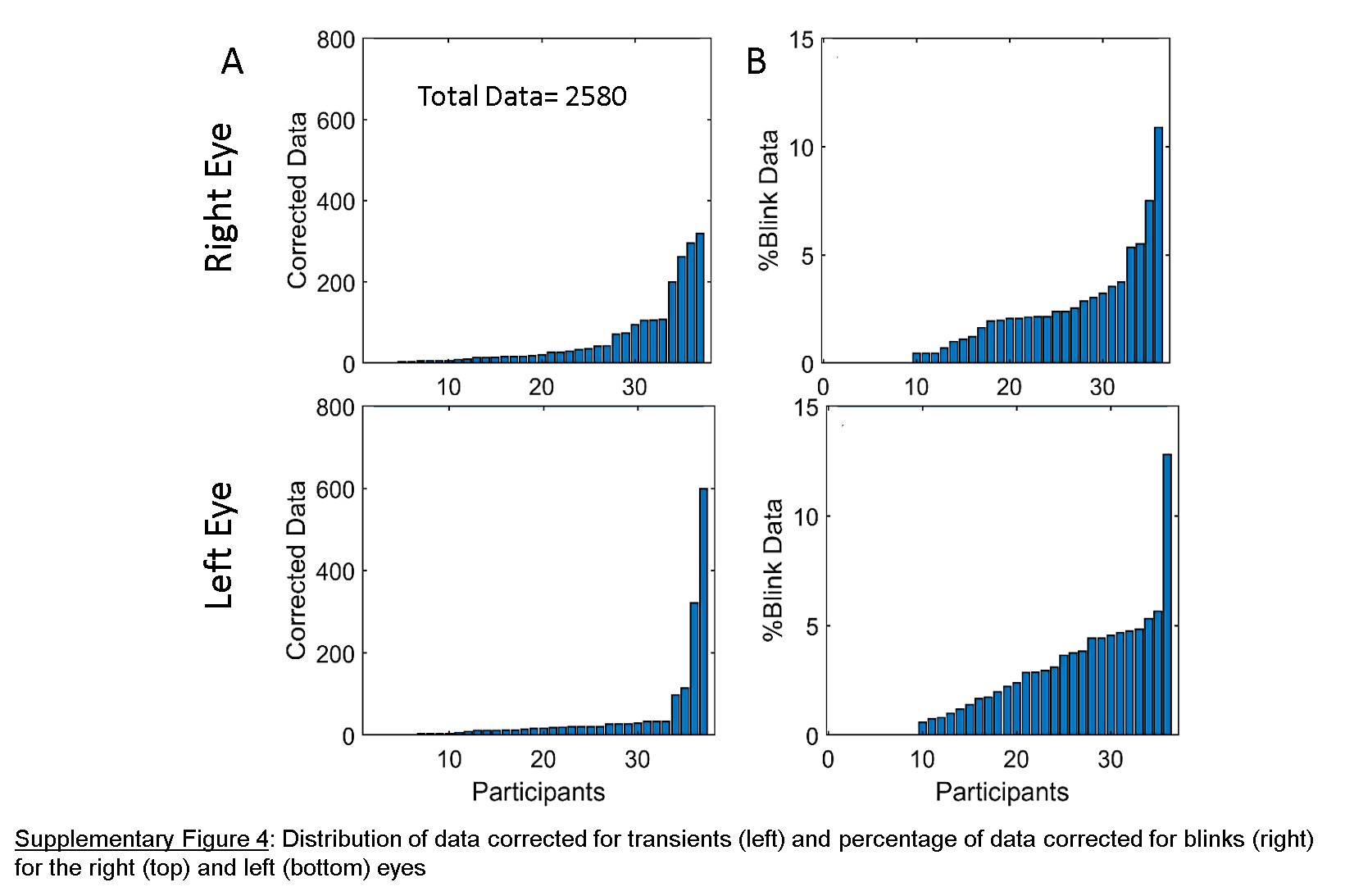

Supplement: Supplementary file 1 [file vision-08-00017-s001.zip › Supplementary Figure S4.png]

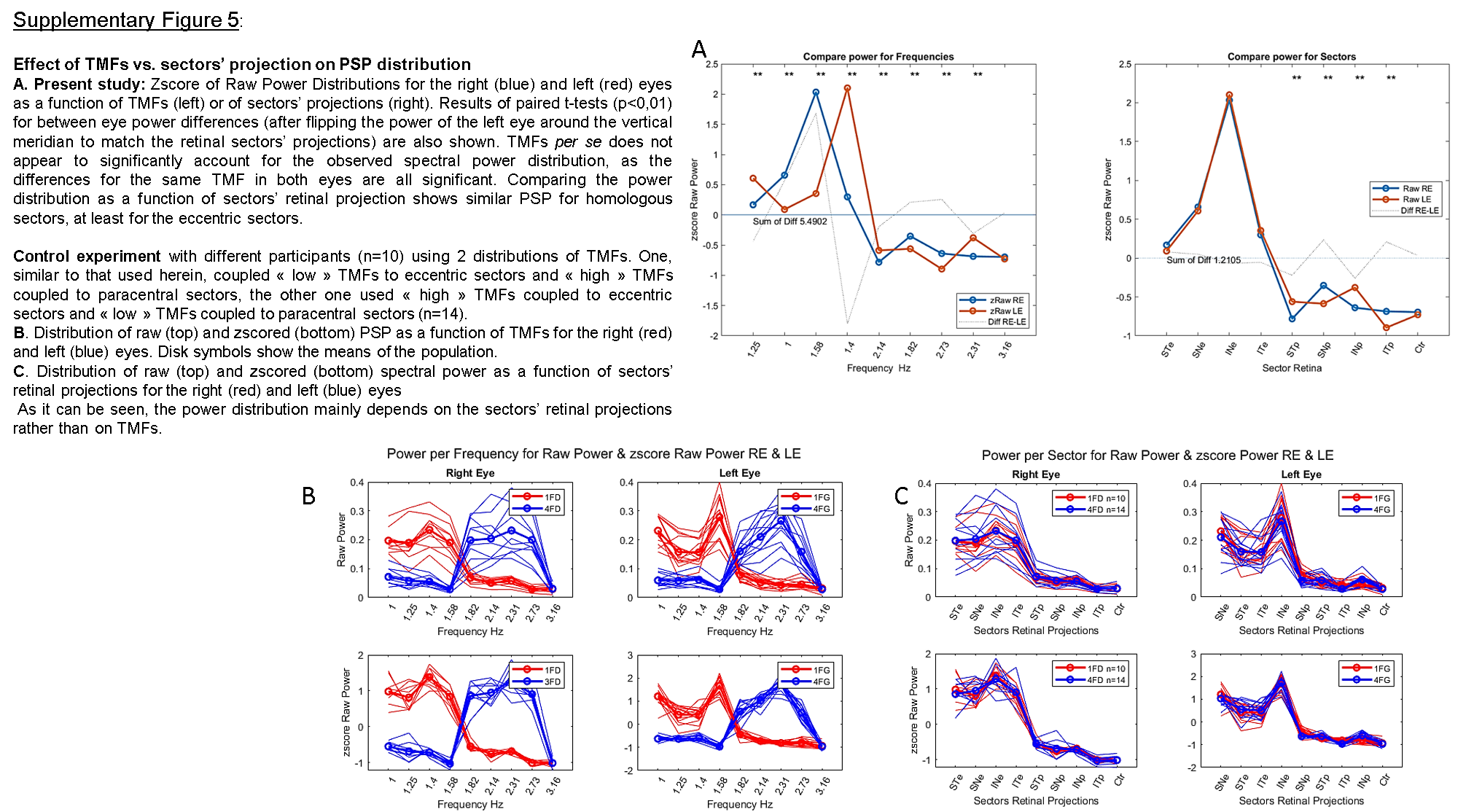

Supplement: Supplementary file 1 [file vision-08-00017-s001.zip › Supplementary Figure S5.png]

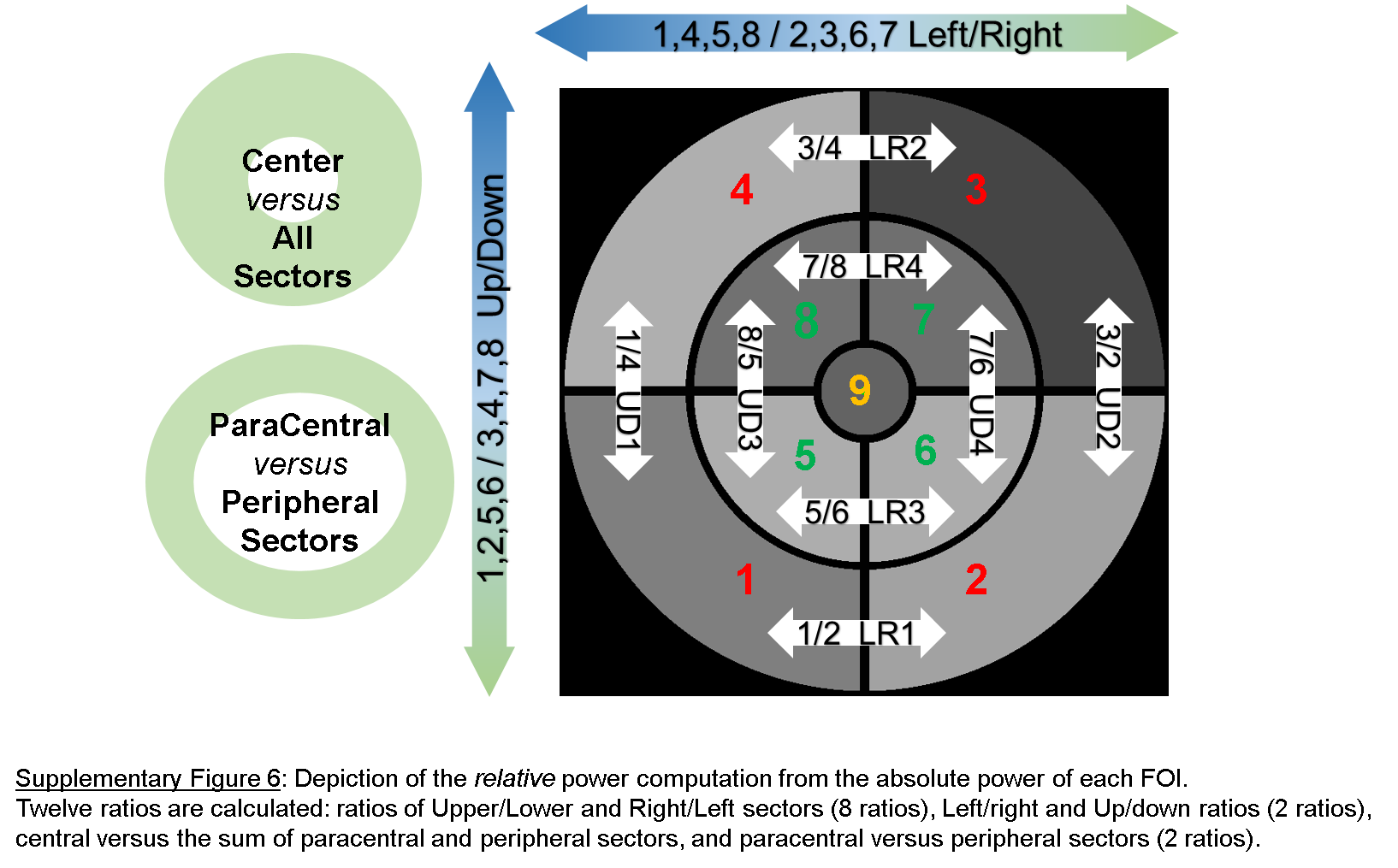

Supplement: Supplementary file 1 [file vision-08-00017-s001.zip › Supplementary Figure S6.png]

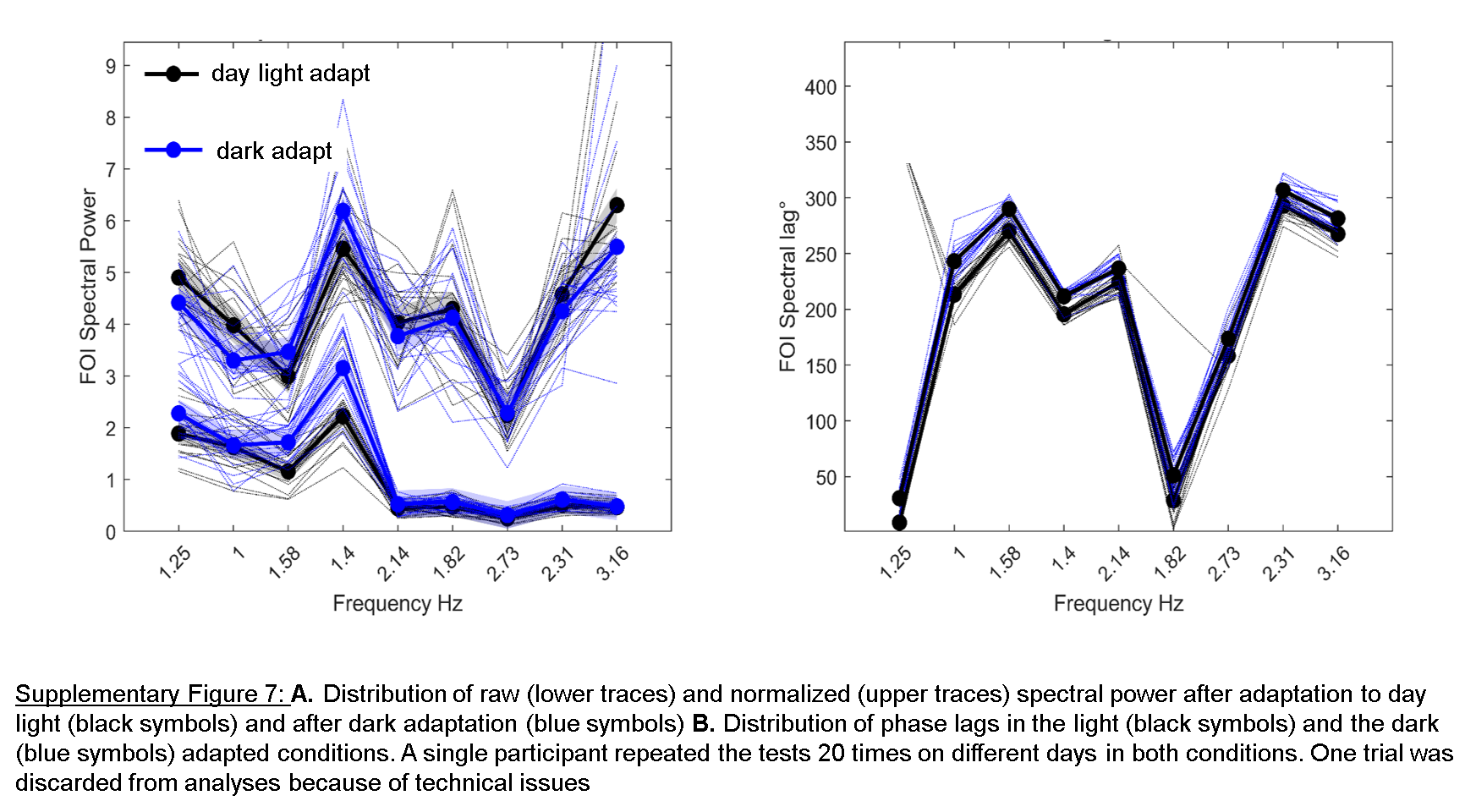

Supplement: Supplementary file 1 [file vision-08-00017-s001.zip › Supplementary Figure S7.png]

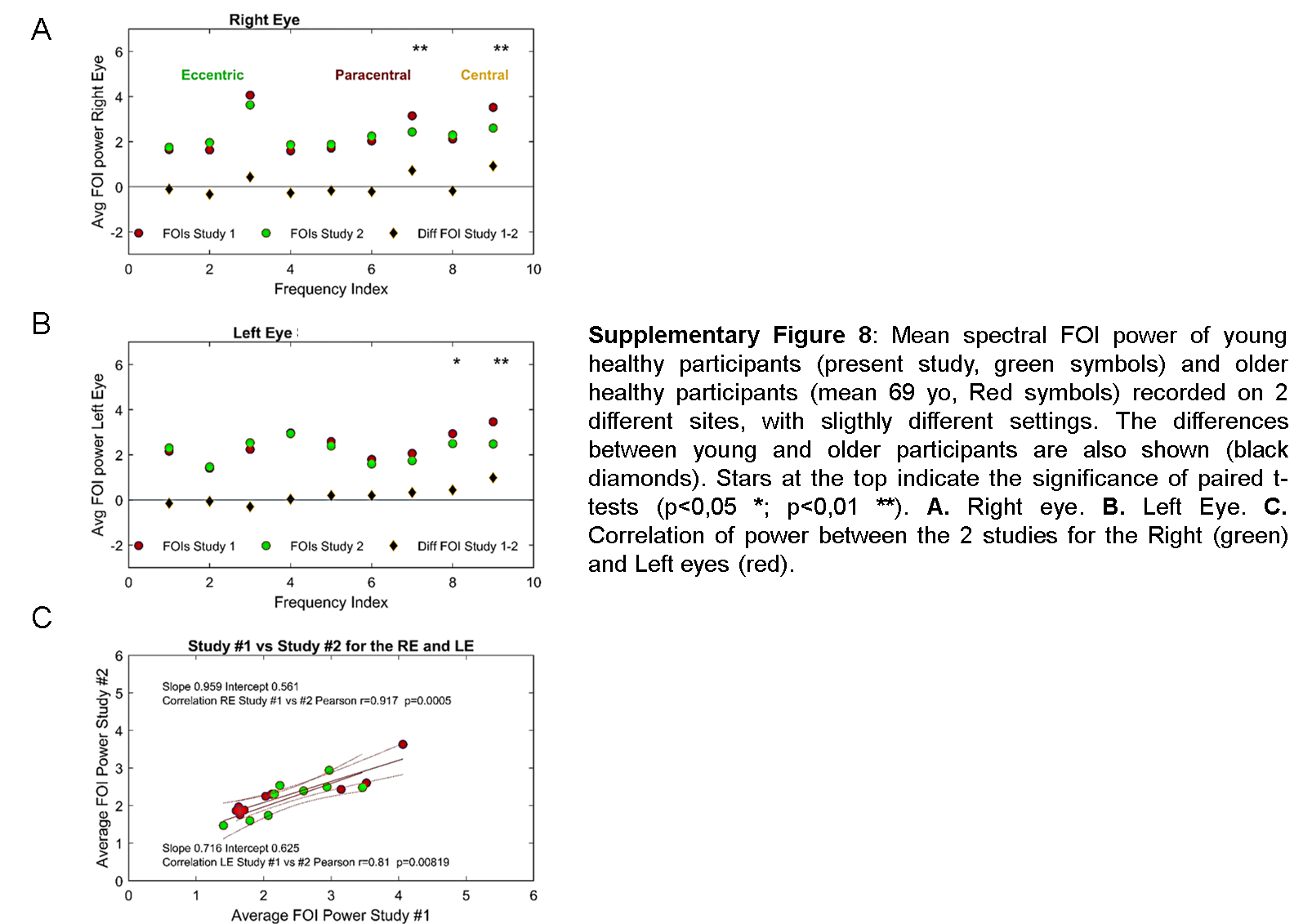

Supplement: Supplementary file 1 [file vision-08-00017-s001.zip › Supplementary Figure S8.png]

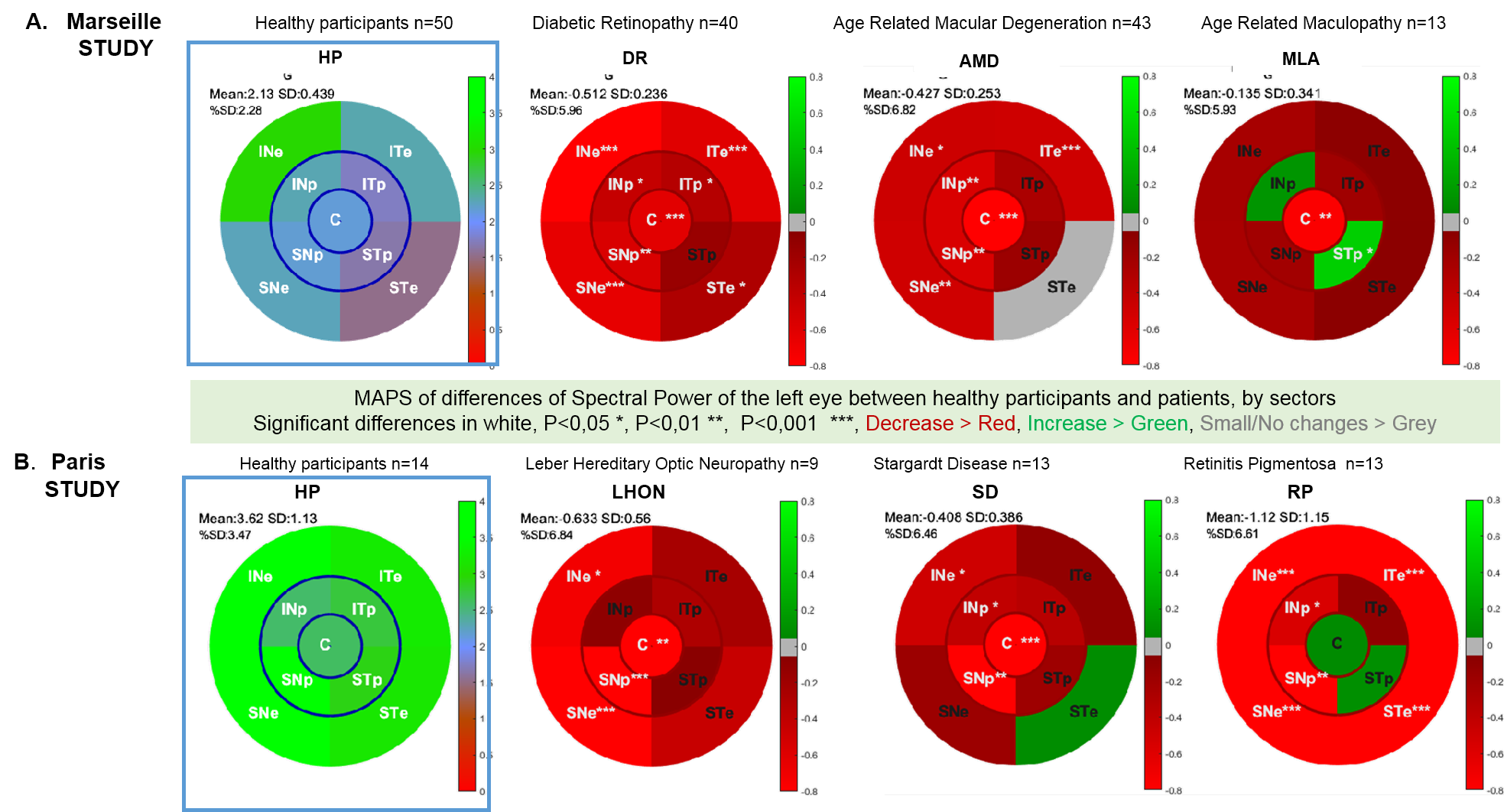

Supplement: Supplementary file 1 [file vision-08-00017-s001.zip › Supplementary Figure S9.png]
